# Supplementary material for: Extreme differences between human germline and tumor mutation densities are driven by ancestral human-specific deviations
Source: Nat Commun. 2020 May 19;11:2512. doi: 10.1038/s41467-020-16296-4 (PMC7237693; doi:10.1038/s41467-020-16296-4)
Supplement: Supplementary file 1 — Supplementary Information [file 41467_2020_16296_MOESM1_ESM.pdf]

Supplementary Information for:

Extreme differences between human germline and tumor mutation densities are driven by ancestral human-specific deviations

Heredia-Genestar et al.

Table of contents

Supplementary Notes: 3
Supplementary Note 1: 3
Supplementary Note 2: 9
Supplementary Note 3: 17
Supplementary Note 4: 24
Supplementary Note 5: 29
Supplementary Note 6: 34

Supplementary Tables: 44
Supplementary Table 1: 44
Supplementary Table 2: 45
Supplementary Table 3: 46
Supplementary Table 4: 47
Supplementary Table 5: 48
Supplementary Table 6: 50
Supplementary Table 7: 51
Supplementary Table 8: 52
Supplementary Table 9: 53
Supplementary Table 10: 54
Supplementary Table 11: 55
Supplementary Table 12: 56
Supplementary Table 13: 57
Supplementary Table 14: 58
Supplementary Table 15: 60
Supplementary Table 16: 61
Supplementary Table 17: 62
Supplementary Table 18: 63
Supplementary Table 19: 64
Supplementary Table 20: 65
Supplementary Table 21: 66
Supplementary Table 22: 67
Supplementary Table 23: 68
Supplementary Table 24: 69
Supplementary Table 25: 70
Supplementary Table 26: 71
Supplementary Table 27: 72
Supplementary Table 28: 73

Supplementary Figures: 74
Supplementary Figure 1: 76
Supplementary Figure 2: 78
Supplementary Figure 3: 80
Supplementary Figure 4: 82

Supplementary References: 83

Supplementary Notes:

Supplementary Note 1:

Data processing

Sample selection

We used 59 chimpanzees (10 *Pan troglodytes ellioti*, 19 *Pan troglodytes schweinfurthii*, 18 *Pan troglodytes troglodytes*, and 12 *Pan troglodytes verus*), 10 bonobo (*Pan paniscus*), and 43 gorillas (27 *Gorilla gorilla gorilla*, 1 *Gorilla gorilla diehli*, 7 *Gorilla beringei beringei*, and 8 *Gorilla beringei graueri*) from the Great Ape Genome Project[1,2]. The gorilla sample Serufuli was excluded because the sample uploaded to the Sequence Read Archive was malformed. We also used the whole release dataset of human samples from the 1000 Genomes Project[3] (1kGP), 50 human samples (24 African, 26 non-African) from the Simons Genome Diversity Panel[4] (SGDP50) and 2,583 human tumor samples (selected from 2,583 different individuals) from the Pan-Cancer Whole Genome Analysis Project[5] (PCAWG, referred as Tumor).

Mapping

We mapped the human (SGDP50) and Non-Human Great Ape (NHGA) samples using BWA MEM[6] to the human reference genome hg19. For the type of analyses we will perform (mutation density of windows across the genome), we require all datasets to use the same reference genome. As we are interested, not only in generating high-quality variant calls, but also in their distribution across the genome, we chose to map all samples directly to the human reference instead of relying on the liftOver tool to convert the genomic coordinates from one reference to another, as liftOver transformation can leave artifactual gaps of low (or high) mutation density in the

destination reference. We chose to use the human reference instead of each species own reference to be able to jointly compare Non-Human Great Ape (NHGA) and human samples, and to take advantage of the more complete human gene models and resources available.

When mapped to the human genome, chimpanzee and bonobo samples cover on average 90.93% of the non-N human reference with good mapping quality and coverage. Gorilla samples cover 90.42%. In order to check how this compares with human samples mapping against their own species' genome, we mapped 10 human germline samples from the PCAWG project[5]. Human samples cover 95.40% of the human reference. These human samples are at higher coverage than the great ape samples (mean depth of coverage, humans: 38.70x; chimpanzee & bonobo: 25.59x; gorilla: 19.89x). To test if this difference in mapping is due to the different coverage, we downsampled these human samples to the median coverage of chimpanzee samples (23.7x) and they mapped 95.11% of the human reference. This shows that the difference in mapping is not caused differences in coverage but by the intrinsic sequence divergence between different great ape species.

The human genome has many repetitive regions. These regions are difficult to map to, even for human samples. We filtered them out to avoid erroneous variant callings from mismapped or collapsed reads (see sections "mappability" and "copy-number"). When these regions are removed, chimpanzee and bonobo samples map to 97.57% of the filtered human reference and gorillas 96.76%, much closer to that of human samples (high-coverage human samples: 98.72%; chimpanzee-level-coverage human samples: 98.36%).

Variant Calling

We called variants on each sample individually using the GATK-3.7 Haplotype Caller[7] and joined all samples within a species using GATK GenotypeGVCFs. On this joint dataset, we applied several filters to remove miscalled variants caused by sequencing errors and potential artifacts. The filters applied were adapted from the GATK best practices guidelines [8,9] and forums (https://gatkforums.broadinstitute.org/gatk/discussion/2806/howto-apply-hard-filters-to-a-call-set).

Variants removed:

- For all variant types:
  - More than 3 variants called within a 10 bp window
  - All samples total depth of coverage < 4*number of samples
  - Individual genotypes with coverage < 4
  - Genotyping quality < 50
  - Quality by depth < 10
- SNV only:
  - Mapping Quality < 40
  - Mapping Quality Rank Sum < -12.5
  - Read Position Rank Sum < -8
  - Fisher Strand Test > 60
  - Strand Odds Ratio > 4
- InDels only:
  - Read Position Rank Sum < -20
  - Inbreeding Coefficient < -0.8
  - Fisher Strand Test > 200
  - Strand Odds Ratio > 10

After that, we used VCFTOOLS[10] to filter out non-biallelic variants (variants with 2 or more alternative alleles) and variants with more than 20% missing genotypes in the samples of a given dataset (chimpanzee+bonobo, or gorilla).

Finally, to minimize the number of variants erroneously called due by mismappings, collapsed duplications or other events, we removed also:

- Variants where heterozygous samples represented more than 80% of all calls.
- SNV within 5bp of an InDel.
- InDels within 5bp of another InDel.
- Regions where the human reference has poor mappability (see section “mappability”).
- Regions where > 25% of the samples of a great ape species were not deemed callable by GATK CallableLoci (either by too low coverage, too high coverage, or poor mapping quality).
- Copy Number Variable regions in great ape lineages (see section “copy-number”).

Mappability

The human reference contains many repeated regions and regions with high homology that could lead to mismapping of sequence reads. In order to minimize the mapping errors caused by these, we used the SNPable regions software (http://lh3lh3.users.sourceforge.net/snpable.shtml) to filter out these non-uniquely mappable regions. To do this, we split the human reference into 35-bp overlapping k-mers, mapped those k-mers back against the same human reference, and kept only those positions of the reference where the majority of overlapping reads mapped uniquely and without mismatches. This resulted in a list of uniquely mappable regions that cover 80.22% of the non-N human reference genome.

Copy-Number

In order to avoid erroneous variant calls from collapsed duplications, we used previously published Copy-Number estimates from great ape samples[11]. We used the LiftOver tool[12] to convert the coordinates to the hg19 reference. We filtered out a total of 134Mb of sequence in regions defined as fixed duplications in human, chimpanzee, bonobo or gorilla samples, as well as those regions copy-number-variable in at least two samples in any of these species.

Variant Calling Results

Variant calling resulted in 52.8M SNVs and 1.6M InDels called in the chimpanzee+bonobo dataset (22.2M SNVs and 0.8M InDels per sample on average), and 41M SNVs and 1.6M InDels called for gorilla samples (30.5M SNVs and 1.3M InDels per sample on average). These samples are NHGA mapped to the human reference, and these numbers include all positions fixed in NHGA but different from the human reference (and thus, derived in either the human or the NHGA branches). If we take into account only non-fixed variants, the average number of segregating sites per sample is 5.9M SNVs and 245k InDels in the chimpanzee+bonobo dataset, and 5.8M SNVs and 271k inDels in the gorilla dataset.

When analyzing each NHGA subspecies individually, we observe that NHGA samples have an average number of SNVs much higher than human samples. This is caused by between-subspecies differences, as the average number of heterozygous sites per sample of each subspecies is much closer to the human average. This number of heterozygous sites per sample can be used as an estimator of population diversity. The results of our calling show chimpanzee between-subspecies estimates similar to those reported in the original manuscript[13] where these samples were mapped to the chimpanzee reference (Average count of heterozygous SNV per sample: *Pan*

paniscus: 1,182,432; *Pan troglodytes ellioti*: 2,013,762; *Pan troglodytes schweinfurthii*: 2,477,963; *Pan troglodytes troglodytes*: 2,999,398; *Pan troglodytes verus*: 1,294,985; *Gorilla beringei beringei*: 1,255,272; *Gorilla beringei graueri*: 1,294,715; *Gorilla gorilla gorilla*: 2,984,205; *Homo sapiens*: 1,669,533).

Calculation of mutation density

We divided the autosomes into 1Mbp overlapping windows (with an overlap of 50% or 500kb). We counted the number of SNVs in each windows (segregating sites in the human, chimpanzee and gorilla population -or germline- datasets; and somatic mutations in the tumor dataset). Mutations present in more than one sample were considered as a single mutation event in the germline datasets, but independent mutation events in each sample in the tumor somatic dataset. We divided this resulting number by the fraction of the window passing our filtering, giving a measure of mutations per 1Mbp. Removing recurrent events from the tumor dataset had no effect in the results of the analyses.

Supplementary Note 2:

Robustness of the results

Effect of the filtering in the windows

The objective of the filtering we used (mappability + callability + copy number) is to analyze only regions of the genome where human and NHGAs have a similar ability to map and call variants. This allowed us to reduce the bias in mutation density estimates in different species. Regions with low homology between humans and NHGAs would show extremely low mutation density in chimpanzee and gorilla because we would not be able to call variants there. The mappability filter has the biggest effect in the global filtering, where 74.07% of the whole genome (including N sites) passes it. The CNV filter removes an additional 1.55% of the genome, and the callability filter of >75% of the chimpanzee+bonobo, and gorilla samples, removes an additional 2.69% of the genome, leaving a total of 69.83% of the genome passing all filters.

Not all the genome is affected equally by this filtering. When dividing the autosomes into 1Mbp windows, we observe a bimodal distribution: some windows pass 0% of our filtering (mainly centromeres, telomeres, and the regions adjacent to them), while most windows pass >50% of our filtering (most passing 75%~90%) (**Supplementary Figure 1**). We set an arbitrary limit of 50% for the windows to analyze, leaving 5,040 windows out of 5,776 for our analysis. The differential distribution of mutation density in human, NHGAs, and tumor is preserved when using higher values for the filtering threshold (**Supplementary Table 3 & Supplementary Figure 1**). When using a filtering threshold of 50%, the human datasets have almost no correlation with the fraction of the window passing our filters (window ratio), while the great ape samples show a positive correlation (as expected because one component of this filtering is calculated from these samples). Surprisingly, the tumor dataset shows a strong negative

correlation with the window ratio that might be mediated by GC-content or CpG-content (**Figure 2d & 3a, Supplementary Figure 3, Supplementary Table 5**).

When analyzing the effect of the different components of this filtering in the distribution of mutation density, mappability has a high effect across all windows, with slightly more effect in windows with low mutation density in all species or with higher mutation density in humans than NHGAs. Callability has a lower effect than the mappability filtering. We would expect the callability filtering to affect more windows with low mutation density in NHGAs (as we would not be able to call variants in them) while being randomly distributed in human (as they should not affect the human genome). But the results show that NHGAs callability has a strong effect only in the windows more densely mutated in human, independently of the density in NHGAs. This suggests these windows have been somehow recently innovative in humans, and these differences cause the mapping problems in NHGA samples. The CNV filtering does not affect differently windows more highly mutated in one species than the other (data not shown).

We compared the mutation density in both the fraction of each window passing and failing the different components of the filtering. We observe that NHGAs have much lower mutation density in the fraction of the windows failing their own species callability filtering, than in the fraction of the window passing all filters. Meanwhile, in both humans and tumors, the mutation density is similar in both the passing fraction and the fraction failing callability in any NHGA species. The CNV filtering has almost no effect in humans, while filtering out in NHGAs some regions that have either higher or lower mutation density, putatively duplications and deletions. These results show that these filters applied are necessary to remove problematic regions inside the windows that could alter the average mutation density of the window.

Window Size

We analyzed the distribution of mutation density at different scales using 10Mbp, 100kbp, and 10kbp windows. The correlations between all germline datasets are stable across all window sizes, becoming weaker in 10kbp windows. Tumor has lower correlations with human than with NHGAs across all window sizes (**Supplementary Table 3**). The human-NHGA-tumor pattern can clearly be detected in 10Mbp and 100kbp windows. Using 10kbp windows, this pattern becomes less apparent, but we can observe a clear distribution driven by the higher correlation between NHGAs and tumor (**Supplementary Figure 1**). The two-sided Mann-Whitney U tests of the mutation density in tumor are always significant, either splitting the dataset above/below the diagonal, or by windows with >25% difference in human and NHGAs.

Sample Size

We tested the robustness of the results using subsamplings of the human dataset 1kGP, with the same sample size as the Chimpanzee (69 samples) and Gorilla datasets (43 samples). The samples were chosen aiming to maximize human diversity. We clearly observe similar correlations between the human, NHGAs and tumor datasets (**Supplementary Table 5**), as well as the same pattern in the distribution of mutations where windows with higher mutation density in NHGAs than in humans, have also higher mutation density in human tumors (**Supplementary Figure 1**). This suggests that the different sample size of the datasets is not the cause of the observed association.

Additional human datasets

We tested the robustness of these results using three additional human datasets different than the 1000 Genomes Project. We used the release dataset of the International Cancer Genome Consortium (ICGC) germline dataset[5] (the germline

samples of some of the tumor samples used in this study), with 1,823 samples; the Simons Genome Diversity Panel (SGDP) release dataset[4], with 279 samples; and our own mapping and calling of 50 samples of SGDP (SGDP50) chosen to maximize human diversity. We can clearly observe that using these three human datasets shows similar between-species correlations (**Supplementary Table 5**), and distribution (**Supplementary Figure 1**) than when using the 1kGP dataset.

These three datasets, as well as the tumor dataset, were generated using PCR-free technology, in contrast with the 1kGP and NHGAs datasets. Other studies[14] have shown that PCR-amplification causes a decrease in coverage of GC-poor/AT-rich regions in Illumina reads, and this effect should disappear when using PCR-free technologies. We detect differences in the distribution of coverage between the 1kGP and NHGAs datasets, and the PCR-free tumor, ICGC, and SGDP50 datasets. Having seen that the distribution of mutation density is very similar when using both 1kGP and these three additional human datasets, proves that this differential distribution is robust to different human datasets and it is not dependent on the underlying sequencing technology used.

Chimpanzee reference

To assess the effect of calculating the distribution of mutation density in NHGAs using data mapped to a reference genome different than their own species, we mapped 50 chimpanzee samples (10 bonobos, and 10 samples from each chimpanzee subspecies), and 50 human samples (SGDP50) to the chimpanzee reference PanTro5. We ran the same mapping and variant calling pipeline used for the human reference calling. We applied a similar filtering, calculating the unique mappability of 35-mers against the PanTro5 reference, the callability of ≥75% of the chimpanzee and human samples, and the great apes CNVs using the LiftOver tool[12] to map them against the chimpanzee reference. We used the number of non-fixed segregating sites in both

species to calculate the mutation density in each window. We used LiftOver also to map the tumor dataset to the PanTro5 reference, and calculated the GC-content of each window in the PanTro5 reference (**Supplementary Table 4, Supplementary Figure 1**). We observe the exact same distribution in tumor mutation density and GC-content as in the datasets mapped to the human reference. Windows where chimpanzee has higher mutation density than human, have also higher mutation density in tumors and lower GC-content than the regions with higher mutation density in humans than in chimpanzee. The fraction of the window passing our filtering is no longer associated with low callability in chimpanzee in GC-rich regions, as in the human reference. This proves that this distribution is neither dependent on the reference used to map the samples, nor the fraction of the windows passing the filters.

Variant Frequency

We divided our datasets and calculated the distribution of the mutation density of each window using only variants of a certain frequency and compared it with the mutation density of the tumor dataset (without altering the tumor dataset variant frequency) (**Supplementary Tables 7,8**). We can clearly detect the differential correlations and distribution pattern in both chimpanzee and gorilla when calculating the mutation density using only non-singleton variants, variants with allele frequency > 5%, variants with allele frequency ≤ 10% or variants with allele frequency > 10%. Variants with allele frequency ≤ 5% do show a clear differential pattern when comparing human with chimpanzee, but the pattern is much more diffuse in gorilla, driven by a lower correlation between gorilla and tumor (data not shown).

When using only singletons or doubletons, the distributions become much more diffuse. The differential correlations between tumor and human or NHGAs are still present. However, the correlations of low-frequency variants between human and great

ape datasets become much weaker, removing the original human-NGHA distribution pattern, and, thus, removing the clear diagonal split of the data.

We also removed any shared variant between the human and chimpanzee or gorilla, calculating the mutation density using only variants exclusive of a given dataset (**Supplementary Table 9**). Using only exclusive variants shows clearly the differential pattern, proving that it is not dominated by variants shared between species, but variants that arose in only one of them. The correlation between shared and species-exclusive variants is also similar in the different species (**Supplementary Table 10**).

Previous studies in this field have used human-chimpanzee and human macaque divergence[15–17]. We reconstructed the human-chimpanzee and human-gorilla divergence from population data. We selected all those variants called homozygous for the alternative allele in all samples of a NHGA dataset, that were not found to be segregating in the 1kGP human dataset. We calculated the mutation density of all windows using only these variants (**Supplementary Table 11**). This distribution does not show any clear pattern. The correlation of these variants with all segregating sites is similar in both human and NHGAs. We cannot observe the differential pattern using divergence data. This distribution is heavily affected by the amount of great ape samples used, as new low-frequency variants will only decrease the number of fixed sites (increasing from 50 to 69 chimpanzee samples leaves only half of the fixed sites; data not shown). We can extract no conclusion of this, but it would be worth revisiting once more great ape samples are available and inferences of divergence from population data are more reliable.

This pattern being observable by variants of high frequency, but becoming more diffuse at extremely low frequencies suggests that there is a common origin in the molecular mechanism or selective pressures generating it, before the human-chimpanzee-gorilla

split. This effect can clearly be detected using variants exclusive from the human and great ape branches, showing that the mechanisms generating it have been acting individually in each branch after the species split. These mechanisms have been common between human and chimpanzee until very recently, but diverged earlier in gorilla, shown by the lower effect in the accumulation of new low-frequency variants.

Recent studies[18] have detected differences in the mutation rate in human in extremely rare variants and de novo mutations when compared with variants at higher frequencies in the population. This suggests that the mechanisms leading to mutation rates have evolved recently in the human genome. NHGA populations could be under similar (although different) effects, leading to this higher differentiation in low-frequency variants.

InDels

Previous studies[17] have described a co-localization of insertions and deletions in regions with high substitution rate and high GC-content. We analyzed the distribution of non-fixed InDels segregating in the population of each of our datasets (**Supplementary Table 12**). The density of InDels correlates best with the mutation density of its own species. InDels in human and chimpanzee have a poor correlation with mutation density in tumor, while InDels in gorilla show a moderate correlation with tumor. When comparing the InDel densities between species, all germline datasets have moderate pairwise correlations. Tumor InDels have a weak correlation with human InDels, slightly stronger with chimpanzee, and a moderate correlation with gorilla InDels. Comparing the distribution of human, great ape, and tumor InDels shows a diagonal pattern only in the case of human-gorilla. This pattern also appears, albeit weakly, when comparing InDel density chimpanzee, gorilla and tumor. This suggests that this co-localization of InDels in gorilla and tumor is either specific from the gorilla

lineage or changed towards the current human distribution in the branch of the common ancestor between human and chimpanzee. Another possible explanation could be that the higher divergence time between human and gorilla makes some regions prone to erroneously call InDels instead of more complex structural events. If these regions were to be more prone to a certain degree of structural variation in the human-chimpanzee branch, they could imply also a tendency towards InDel mutations in tumors.

Supplementary Note 3:

Additional datasets

Tumor types

To determine if this differential mutation density pattern we detect is caused by specific tumor types, we divided 2,581 tumor samples with known histology into 37 histological categories (as reported in the original dataset)[5] and calculated the window mutation density on each of them. We observe clear differences in the correlation of human and NHGAs with each of the tumor types (**Supplementary Table 14**). Although there is variability between different tumor types, with some tumors having a stronger correlation with the germline human mutation density than others, these human-NHGA-tumor differences in correlations are present in virtually all tumor types with enough sample size and number of SNV.

Tumor mutation density is affected by the chromatin status of the tissue of origin[19]. Among the tumor types with more mutations in the dataset (> 1M SNV), all have very high correlations (**Supplementary Table 15**). The only exceptions to this are Skin-Melanoma and Breast-Adenocarcinoma. The different mutation distribution in Skin-Melanoma might be caused by a very strong contribution of mutational signatures SBS7a-d caused by UV-light exposure[20]. In addition, UV-damage in melanoma cells is enriched in the nuclear periphery regions[21]. To the best of our knowledge, the differential distribution in Breast-Adenocarcinoma has not been previously described. We hypothesized that these differences might be driven by the defective *BRCA1/2* repair, represented by the mutational signature SBS3. We divided the Breast-Adenocarcinoma samples in two groups of samples presenting or not signature SBS3[20]. We observed no difference in the correlations of both groups, suggesting that

this different mutation distribution in Breast-Adenocarcinoma is not driven by signature SBS3.

The PCAWG dataset does not include any cancer originating in germinal cells. Interested in testing if human germinal cell tumors had mutations distributions more similar to the human population than other tumors, we obtained data from two cancer types: Spermatocytic Tumors[22], and Nonseminomatous Testicular Germ Cell Cancer[23]. Spermatocytic tumors have been described to have a mutation profile similar to human variability[22] (a combination of SBS1 + SBS5). We combined both datasets and correlated them with humans and NHGA populations. Unfortunately, due to the small sample size and mutation rate of these tumors (8 samples, 2,710 SNV passing our filters) we obtained no correlation in their mutation distribution with any of the datasets. Further studies of these tumor types will be needed to properly assess the mutation distribution in germ cell cancers.

Sex and age

The great ape datasets are female-biased (Male/Female samples, 1kGP: 1,233/1,271; chimpanzee: 23/45; gorilla: 10/33; tumor: 1,420/1,160). However, the number of heterozygous variants present in samples of a population is similar between sexes (two-sided Mann-Whitney U test number of autosomal heterozygous sites in males vs females within a subspecies p-value > 0.05 in all NHGA subspecies). In contrast, tumors can be sex-specific, have different incidences by sex, or have different mutational loads by sex[24]. We compared the mutation distribution in human and NHGA with male and female tumor samples. We can clearly observe the differential distribution pattern and the NHGA-tumor correlations are higher than the human-tumor correlation in both males and females, when using all tumor types and when removing

sex-specific cancer types (breast, ovary, uterine, cervix, and prostate) (**Supplementary Table 14**).

Both cancer incidence and its mutation load increases with the patient's age[25–27]. Age also has an effect on the mutation load of blood samples[28], although small (~1,000 SNV at age 60) when compared with the total number of mutations[29].

We retrieved the available phenotypic information from the PCAWG samples and divided the cancer dataset into 3 age groups: <30, 30-59, and ≥60. As the age information was not complete for all tumor samples, we considered the sample's age to be "age at recruitment", "age at last checkup", or "age at diagnosis" in that order. No age information was available for the human and NHGA population datasets. All 3 cancer age groups show a differential distribution pattern and NHGA-tumor correlations are higher than the human-tumor correlation (**Supplementary Table 14**).

Archaic hominids

The analysis of mutation distribution in archaic hominid samples can help us narrow down the time point in human evolution in which the differential human-NHGA-tumor pattern arose. We downloaded the variant calling files from 3 high quality archaic hominids: Altai and Vindija 33.19 Neanderthals[30,31], and Denisova[32]. We downloaded the callability mask of all 3 samples (from http://cdna.eva.mpg.de/neandertal/Vindija/), and intersected it with our previous filters. This left a total of 1,433 Mbp of high quality sequence to analyze, and 4,130 1Mbp windows where ≥50% pass the filtering. Using this new common filtering, we calculated the mutation density in 1Mbp windows in a dataset composed by all 3 archaic samples together, as well as our previous human, chimpanzee, gorilla, and tumor datasets (**Supplementary Table 13, Supplementary Figure 1**). We observe an intermediate correlation between modern humans and

archaic hominids mutation density, similar to the modern humans-chimpanzee correlation. This might be caused by real differences in mutation density, or by the extremely small sample size (3 samples) of the archaic hominids. In contrast, the correlation between archaic hominids and tumor mutation density is very low, similar to the human-tumor correlations, and much smaller than the tumor-NHGA correlations. When comparing the ranked mutation density of archaic, we observe a random distribution of tumor mutation rates when comparing modern humans and archaic hominids. Comparing NHGAs with archaic hominids shows a pattern more similar to the human-NHGA-tumor distribution. This pattern does not reproduce the diagonal pattern previously seen and is dominated by the higher tumor-NHGAs correlation, probably caused by the small sample size of archaic hominids. This suggests that the mechanisms causing the differences between humans and NHGAs have been active since well before the split between humans and Neanderthals and Denisovans. These results are conditioned by the extremely small sample size of the archaic dataset, as well as the sequence fragmentation. The absence of a deep tail of CpG-rich regions in archaic-NHGA comparisons suggests that the CpG>T mutation acceleration might be more recent than the human-Neanderthal split. However, this observation is far from definitive, as a large number of CpG>T mutations might have been removed from the archaic dataset as part of the ancient DNA damage filtering process. We found no association with regions of Neanderthal introgression in modern humans (data not shown).

Great ape subspecies

We calculated the mutation density of each NHGA subspecies separately, analyzing only segregating sites in the subspecies while removing subspecies-specific fixed variants, to assess if specific populations drive this human-NHGA-tumor pattern observed. We observe that, the less diverse subspecies *Pan paniscus* (bonobos), *Pan*

troglodytes verus (western chimpanzees), *Gorilla beringei beringei* (mountain gorillas), and *Gorilla beringei graueri* (eastern lowland gorillas), have lower correlations (**Supplementary Table 5**) with human mutation density than the most diverse subspecies (*Pan troglodytes troglodytes* (central chimpanzees), *Pan troglodytes schweinfurthii* (eastern chimpanzees), *Gorilla gorilla gorilla* (western lowland gorillas)). This lower correlation is not mediated by their lower sample size, as subsets of 5 *Pan troglodytes troglodytes* or 5 *Gorilla gorilla gorilla* samples have higher correlations. These low-diversity subspecies also have lower correlations with the mutation density in tumors than the more diverse subspecies, but the correlation between great ape subspecies and tumor is always higher than between different human datasets and tumor. Low-diversity subspecies also have lower correlation with GC-content and the fraction of the window passing all our filters.

We compared each of these subspecies against a subset of humans of the same sample size. We selected different subsets of human samples of African origin to maximize human diversity, from the previously used subsets of the 1kGP dataset. We can clearly reconstruct the human-NHGA-tumor pattern when using high-diversity subspecies, even when using only 5 samples in the NHGA dataset. Low-diversity subspecies, though, have a much more random distribution of the mutation rate in tumor, with no clear diagonal pattern, although they have a slightly higher correlation with tumor than humans (**Supplementary Figure 2**). We hypothesize that this diagonal-split distribution is caused by comparing a high-diversity dataset against a low-diversity dataset, regardless of the low-diversity dataset being human samples or not. If this were to be true, we should be able to reconstruct the diagonal split by comparing high-diversity NHGAs with low-diversity NHGAs. When we do this, we observe a clear horizontal split distribution of the mutation rate of tumor, mediated by the higher correlation of tumor with the high-diverse datasets, but we were not able to reconstruct the diagonal split that we see when comparing high-diversity NHGAs with

humans. Comparing two low-diversity subspecies shows no clear pattern, while comparing two high-diversity subspecies shows tumor mutation density being higher in the windows with higher mutation density in both subspecies. It is worth noting that, in none of the tested cases, we were able to invert the human-NHGA-tumor pattern and detect higher mutation density in tumors in windows with higher mutation density in humans than in NHGA, even when comparing humans with NHGA subspecies with lower diversity than humans.

Orangutan

We obtained 24 orangutan whole-genome samples[1,33], and mapped them to the human reference hg19 using the same methods used for the other datasets. 2,005,340,322bp in the autosomes passed the joint filtering after the orangutan callability filter was applied (47Mbp less than the filtering using only chimpanzee and gorilla samples).

Orangutan samples, like other NHGA, present high correlations with tumor (Pearson's R=0.55). However, we can also observe that the correlation between human and orangutan is weaker (R=0.43) than with other NHGA. This effect follows a trend where the correlations between humans and NHGA decrease as the evolutionary distance increases (**Supplementary Table 6**). The human-NHGA-tumor diagonal pattern can also be observed in orangutan, although much weaker, caused probably by the lower human-orangutan correlations (two-sided Mann-Whitney U test p-value=4.7e-78) (**Supplementary Figure 1**). Although this diagonal pattern is weaker in orangutan than in gorilla or chimpanzee, when analyzing mutations at CpG>T and non-CpG sites, orangutan correlations show similar patterns as the other NHGA species studied, with a high human-orangutan correlation in CpG>T mutations, and weaker human-orangutan than orangutan-tumor correlations in non-CpG sites (**Supplementary Tables 19-20**). Orangutans, like other NHGA, also have strong CpG>T/non-CpG intra-

species correlation (R=0.71). All in all, this suggests that the described patterns observed in chimpanzee and gorillas are also present in orangutan samples. However, the diagonal pattern appears weaklier due to a lower human-orangutan correlation.

Supplementary Note 4:

Genomic Features

Recombination

Recombination rate has previously been correlated with high mutation density [16,34–36] but has shown to have poor correlation with mutations in tumor[37]. We compared male, female, and average human recombination maps[38], human hotspots of Double Strand Breaks (DSB) in PRDM9 binding sites[39], chimpanzee (*Pan troglodytes verus*) recombination maps[40], and chimpanzee (*Pan troglodytes ellioti*), bonobo and gorilla (*Gorilla gorilla gorilla*) recombination maps[41] (**Supplementary Table 16, Supplementary Figure 3**). All measurements of high recombination rate tend to locate in windows with high mutation density in both humans and NHGA, with no big differences between species.

Chromatin State

Previous studies have analyzed the effect of a wide variety of genomic features in the variation of mutation density in the human germline and in human tumors [37]. Conservation and recombination rate were described as the two main drivers of variation in distribution in the human germline, while a wide range of histone marks affecting the chromatin state had very weak correlations with germline mutation density distribution. In contrast, tumor mutation density showed very strong positive correlations with histone modifications associated with closed chromatin, and very strong negative correlations with modifications associated with open chromatin, suggesting that mutation rates in tumor are higher in regions of closed chromatin, probably driven by a poor accessibility to the mismatch repair machinery. Further studies have shown that this correlation between chromatin state and tumor mutation density is more strongly correlated when matching the cell type of both the tumor and

the histone marks used[19]. This strong correlation between the cell-of-origin chromatin structure and mutation distribution allows even to distinguish the tissue of origin of metastatic tumors[42].

We reproduced the analysis of Schuster-Böckler, B., Nature 2012 using our human, tumor and NHGA datasets. We used the same list of histone marks, chromatin modifiers, and genomic features from the original article using the same data sources and processing (**Supplementary Table 16**), except an updated measurement of conservation using 100 vertebrates[43] instead of 29 mammals and updated versions of the ENSEMBL gene lists. The results using our datasets match perfectly the results for 1Mbp windows of Figure 1 and Supplementary Figure 2 from Schuster-Böckler, B., Nature 2012 (**Figure 2**). The correlations of both tumor and human germline mutation densities with genomic features show the same trends as the original article. Tumor has a strong positive correlation with closed chromatin features and a strong negative correlation with open chromatin features, while human germline has a strong positive correlation with recombination rate and H3K27me2 (unexplained in the original article), a strong negative correlation with conservation, and lower correlations with the rest of features. The correlations with mappability of our dataset are biased by our previous mappability filtering of all the datasets. We also analyzed the mutation density of human-chimpanzee and human-gorilla divergence measured as fixed SNV called in the great ape datasets and not present in the human population. This measurement shows differences with the original article human-chimpanzee divergence, but all the correlations are very weak and in the same direction as the human germline (data not shown). Finally we analyzed the correlation with genomic features of chimpanzee and gorilla mutation densities. Both chimpanzee and gorilla, like human germline, have a strong positive correlation with recombination rate and H3K27me2, and a strong negative correlation with conservation. Strikingly, both chimpanzee and gorilla have

moderate to strong correlations with the rest of histone modifications, in the same direction as tumors, although weaker.

We downloaded ChromHMM chromatin state partitioning data from ENCODE[44] in lymphoblastoid cell-lines and compared the overlap of the different chromatin states with the human-NHGA distribution of mutation density. We observe a very similar pattern as previously described, where regions with higher mutation density in NHGAs than in humans co-localize with closed-chromatin features (**Figure 2**).

GC-content

We calculated GC-content as the fraction of the base pairs in a window passing our filters and deemed to be either Guanines or Cytosines in the human reference Hg19. We observe a clear differential distribution with GC-content being higher in windows whit higher mutation density in human than in NHGAs (**Figure 2d**), This distribution is consistent when mapping the samples against the chimpanzee reference panTro5 (**Supplementary Figure 1**). It has been previously reported[15,16] that human mutation density (calculated as human-chimpanzee or human-macaque divergence) has a non-linear association with the GC-content of the underlying sequence at 1Mbp scale. We do detect this U-shaped distribution, but it is driven by a very small number of windows with extremely high GC-content and CpG-content. When we plot this inverting the axes and using the ranked mutation density (which has a similar effect to binning windows by their mutation density), humans show a clear tendency of increasing GC-content in high mutation density windows, while NHGA have a slightly decreasing tendency, and tumor a strong decreasing tendency (**Supplementary Figure 3**). The different NHGA subspecies show similar tendencies with GC-content. When comparing the mutation density of high diversity and low-diversity subspecies, we do not observe a clear pattern in the distribution of GC-content as in the case of comparisons with human

(**Supplementary Figure 3**). The association of GC-content and the ranked mutation density of NHGA subspecies is not different from that of using all subspecies pooled together. This association also holds when using human and chimpanzee samples mapped to the chimpanzee reference panTro5 (**Supplementary Figures 1,3**). GC-content also correlates with the density of protein-coding exons[45–47] (**Supplementary Figure 3**).

CpG sites

CpG hypermutable sites have a mutation rate one order of magnitude higher than other sites in the genome[48–51]. CpG-sites correlate highly with GC-content and genomic and epigenomic features.

We filtered our datasets, selecting only those sites where the reference in human (hg19), chimpanzee (panTro5) and gorilla (gorGor3) has the exact same nucleotide in that site and the adjacent +/-1 base pairs. This allows us to compare mutations at CpG sites without biases caused by CpG>T changes accumulated in the branch of the reference used and fixed in their genome. CpG sites show the same differential distribution as GC-content (**Figure 2d, Figure 3a, Supplementary Figure 3**), and represent a similar fraction of all SNV in all germline datasets (**Supplementary Table 21**). Tumor has a lower fraction of mutations at CpG sites.

CpG methylation

The methylation status of CpGs has an important effect on their mutability, as methylated CpG sites can undergo spontaneous Cytosine deamination, mutating to a Thymine and leading to a transition. CpG-islands are deemed to have lower mutation density, as they tend to be unmethylated. To analyze if the different association of GC-content with mutation density in humans and NHGAs, we downloaded information of

the methylation status of human CpG sites from the ENCODE project[52], and differences in methylation of CpG sites in human, chimpanzee, and gorilla[53] (**Supplementary Table 16**). We observe that both CpG islands and CpG methylated sites co-localize strongly with high CpG and GC-content in the human reference (**Supplementary Figure 3**). We do not observe any difference in the distribution of CpG sites hyper or hypo-methylated in human and great ape species (data not shown). This shows that these different correlations of mutation density with GC-content are not caused by neither changes in the GC-content of the sequence in great apes nor changes in the distribution of hypermutable methylated CpG sites. One strong caveat of this analysis is that methylation was measured from blood samples in all species. It has been shown that germline progenitor cells, specially testis, show increased mutation at methylated CpG sites[54].

Supplementary Note 5:

CpG>T / non-CpG

Analysis of non-CpG sites

We divided the dataset and compared independently mutations at non-CpG sites, and CpG>T transitions at CpG sites. We corrected the mutation density at non-CpG sites by the non-CpG fraction of the window passing all our filters. Mutation density at non-CpG sites shows between-datasets correlations similar to the correlations obtained when using mutations in all sites, with slightly stronger correlations between human and NHGAs, and between tumor and all datasets, still maintaining their differences (**Figure 3f, Supplementary Tables 17,19**). The comparison of mutation density with the CpG content of the window shows a similar distribution as when using all sites, although both human and NHGAs have (as expected), lower CpG content in windows with higher mutation density. This puts NHGAs at a similar level with tumors, with humans being slightly higher, both when using the ranked (**Figure 3b-f**) or unranked (**Supplementary Figure 3**) mutation density. When comparing human, chimpanzee and tumor, we obtain a similar distribution as when using all sites. Using mutations at non-CpG sites, though, mostly removes the diagonal split pattern seen before and instead shows a more horizontal divide (**Supplementary Figure 3**) like in the case of comparing high and low-diversity NHGAs (**Supplementary Figure 2**). Using non-CpG sites we observe still some structure in the GC-content, unlike in the case of NHGAs subspecies, suggesting that non-CpG, GC-rich sites have increased mutation density in humans. Mutations at non-CpG sites in low-diversity NHGA subspecies show weaker correlations with all datasets (as humans do) than high-diversity subspecies

Analysis of CpG>T transitions

Transitions at CpG sites (xCG>T) when corrected by the size of the window passing all filters is similar to an account of the number of CpG>T mutations in the window. This measure shows extremely high correlations between all the germline datasets (**Figure 3d**) but no correlation with tumor. The relationship between mutation density and CpG in the window (using both ranked and unranked data) is identical in the germline datasets (with more mutated windows having more CpG content), while very different in tumor (windows with more CpG>T mutations tend to have low CpG content, and high-CpG content is present in windows with low mutation density). This difference between germline and somatic datasets can be explained by an increased mutation rate of methylated CpG sites in germline cells[54]. The combination of this distribution of uncorrected CpG>T transitions and mutations at non-CpG sites reconstruct the distribution observed when using all mutations together: human and NHGAs have similar distributions in CpG>T transitions, but different in non-CpG mutations, where NHGAs resemble tumor. The sum of both mutation types gives as a result the distribution seen pooling all samples together (**Figure 3b & Supplementary Figure 3**). Humans have very strong correlations of uncorrected CpG>T transitions with all datasets. However, low-diversity NHGA subspecies have much weaker correlations than both humans and high-diversity NHGA subspecies (**Supplementary Tables 18,20**). This suggests an effect on CpG>T sites of human-specific events.

When correcting CpG>T transitions by the number of CpG sites in the window reference, the pattern inverts, and now all datasets, including tumor show extremely high correlations (**Supplementary Figure 3**). All datasets show a very similar distribution between mutation density and CpG-content, with CpG-rich windows having lower mutation density than CpG-poor windows. The differences in the distribution of CpG>T when correcting or not by the number of CpG in the sequence can be explained by CpG-rich windows having more CpG-islands which tend to be

unmethylated, and thus, do not spontaneously deaminate. CpG-rich windows have more CpG>T transitions in total number, as there are more CpG bases to mutate, but they have less CpG>T mutation rate than windows with less CpG sites[55]. When comparing the relative mutation rates of CpG>T and non-CpG sites (**Supplementary Figure 3; Supplementary Table 24**), we observe that windows in human have a poor correlation between the mutation rate of CpG>T and non-CpG (1kGP R=0.39; 1kGP_69: 0.54; SGDP50: 0.56), while the correlation is much stronger in NHGAs (chimpanzee R=0.67, gorilla R=0.84), and extremely high in tumors (R=0.94). This suggests that the mutation rates of CpG sites and non-CpG sites are decoupled in the human germline. One possible explanation for this effect is a recent population expansion in human, which caused a higher accumulation of the more clock-like CpG>T transitions. Another possible explanation would be a saturation of CpG>T transitions in great ape and tumor genomes, as the number of xCG sites is smaller than any other trinucleotide in the genome and a high number of CpG>T mutations probabilistically lead to repeated mutations in different samples.

Correlation of CpG>T / non-CpG mutations and genomic features

In a previous analysis, we have described how NHGAs had correlations with certain genomic features more similar to tumor than to the human germline ones. Several of these features, like the histone marks H3K36me1 or H3R2me1 are highly associated with CpG sites[56,57]. As we have seen, the mutation densities at CpG and non-CpG sites vary between datasets.

We analyzed the correlation of the different genomic features with the different types of mutations. Uncorrected CpG>T mutations (which have an extremely high correlation between germline datasets, and no correlation with tumor), show high correlations with all high-CpG-content and open chromatin-associated genomic features in germline datasets (similar in all), and moderate to strong negative correlations in tumor. Using

the sequence-corrected CpG>T mutation density (highly correlated in all germline and tumor datasets, and inversely correlated with CpG-content), shows identical strong negative correlations with CpG-associated features. However, when using mutations at non-CpG sites (similar in NHGAs and tumor, and different in human), we observe similar strong correlations of NHGAs and tumor with low-CpG, closed chromatin-associated features, while humans show much weaker correlations in the same direction, albeit stronger than when using all sites (**Supplementary Figure 2**).

Similar as the case of the global mutation density being affected differently by CpG>T and non-CpG mutations, the correlations with genomic features are also affected by both types of mutations. While non-CpG mutations in all datasets correlate with closed chromatin features (strongly in tumor and NHGA, moderately or weakly in humans), CpG>T correlates strongly and positively with open chromatin features in germline datasets. The combination of both effects creates the gradient seen using all variants (**Figure 2a**) where tumors have a strong correlation with closed-chromatin features, NHGAs have moderate correlations, and humans have very weak, non-existent, or are even correlated with open-chromatin features.

Recurrent mutations at CpG sites

It has been previously reported that hypermutable CpG sites are prone to mutations appearing independently in different samples[58,59]. Taking into account that the NHGAs mutation rate per year is 1.5x higher than in humans[60], we hypothesized that the mutation density calculation in NHGAs might be biased by recurrent CpG mutations. We analyzed the number of doubleton variants at CpG sites that are shared between populations or subspecies in all germline datasets (**Supplementary Table 22**). When dividing the datasets into a similar number of groups (otherwise, having more groups would lead to more between-group matches), we observe a similar proportion of SNV

doubletons at CpG sites that are shared between distant populations. This suggests that the effect of recurrent mutations at CpG sites is similar in all datasets. Thus, the effect of CpG>T saturation must be similar in all germline datasets.

Human de novo mutations

De novo mutations offer an unbiased way to assess recurrent CpG>T mutations. We used a dataset of 77k de novo mutations in human trios[61] (after filtering). We used the liftOver tool to map the original dataset to the hg19 human reference.

Using all mutations pooled together, human de novo mutations present a much strong correlation with population-based human germline datasets, weak with NHGAs germlines, and almost no correlation with tumor datasets (**Supplementary Table 23**). Due to the much smaller number of mutations in the de novo dataset, we repeated the correlation analysis using Poison Regression and Negative Binomial Regression, methods better suited to deal with the stochasticity of data based in counts. Both methods showed similar correlations between datasets as the Pearson's correlation.

We divided the human de novo mutations into non-CpG and CpG>T mutations. Non-CpG mutations showed similar correlations as all mutations pooled together (moderate with human, weaker with NHGAs, even weaker with tumors). CpG>T human de novo mutations showed strong correlations with all the germline datasets, and no correlation with tumor (**Supplementary Table 23**). This suggests that, although we detect differences between human de novo mutations and population-based mutations in both humans and NHGAs (and more so in tumor), these differences are not driven by CpG>T mutations, which show similar correlations in the germline of all studied great apes. The observed differences in non-CpG sites may still be caused by the small number of mutations of the de novo dataset. Further analysis would be needed to assess this effect.

Supplementary Note 6:

Trinucleotides

Analysis of Trinucleotides

Previous studies have show that the mutation rate of a nucleotide is affected by the adjacent sequence context[62]. Trinucleotides are the minimum unit used for sequence context analysis, composed by the mutated nucleotide and the adjacent sites at both the 5' and 3' ends. Combined with 3 possible mutations, this gives a total of 192 possible sequences that, once folded by the complementary sequence, leave 96 possible combinations of sequence and mutation.

One major caveat when comparing mutations between close species is the definition of the ancestral and derived alleles. This is extremely important when analyzing trinucleotides, as misidentifying the ancestral allele will classify a mutation as a completely different trinucleotide, biasing our analysis. We filtered the SNV data in all datasets in order to use only a set of high confidence variants using the following criteria:

- The trinucleotide sequence can be mapped from the human hg19 reference to the chimpanzee reference panTro5 and the gorilla reference gorGor5 using the liftOver tool.
- The trinucleotide sequence must be identical for all 3 alleles in all 3 reference genomes (hg19, panTro5, and gorGor5), accounting by strand changes.
- The trinucleotide sequence cannot overlap the borders of our previously defined window filters (all 3 basepairs must be within the part of the window passing our filters).
- The trinucleotide sequence cannot overlap a multi-nucleotide variant (two or more adjacent SNV variants called in the same sample).

- The trinucleotide cannot overlap fixed SNV in the adjacent +1/-1bp in another species.
- The trinucleotide cannot overlap an InDel in the analyzed species.
- The trinucleotide cannot overlap an InDel with frequency ≥50% in the other two species (for example, in the case of analyzing chimpanzee, remove InDels with AF≥50% in human or gorilla).

The resulting subset of high-confidence variants leaves 47,872,324 SNV in the human 1kGP dataset (81.78% of the original dataset), 10,651,708 SNV (73.38%) in the human SGDP50 dataset, 26,871,923 SNV (77.61%) in the chimpanzee dataset, 9,813,306 SNV (63.46%) in the gorilla dataset, and 30,756,260 SNV (90.24%) in the tumor dataset. This filtering affects mostly the NHGA datasets, as they are most distant of the human reference used. In addition, the NHGA reference genomes are built using an individual of a given subspecies, causing misidentifications of the ancestral and derived alleles when compared with the human reference. For example, if an SNV is present only in the central chimpanzee subspecies, there is a chance that this SNV was set as the reference allele in the chimpanzee reference genome, when the rest of chimpanzee subspecies carry the same ancestral allele as the human reference.

This 3-sequence filtering affects more CpG sites (85% passing) than non-CpG sites (95% passing) in humans, caused by the hypermutability of CpG sites, where many of them are mutated and annotated as TpG or CpA sites in other references. This sequence bias is found also in NHGAs, although the difference between CpG and non-CpG sites is smaller (**Supplementary Figure 4, Supplementary Table 25**).

Biased assessment of CpG sites

Our assessment of mutations at CpG sites is, by definition, biased. CpG>T transitions represent 10.6% of all trinucleotide variants in the 1kGP dataset (chimpanzee: 9.9%; gorilla: 10.3%). Once we apply our 3-sequence filtering, they become 9.5% of all passing sites in the 1kGP dataset (chimpanzee: 9.3%; gorilla: 9.8%). Previous de novo mutations in human give CpG>T estimates between ~13%[63], and 16~17%[61,64,65]. This difference could be expected just by the nature of the data used. De novo mutations account for new mutations in each individual, while our analysis of segregating sites in population data masks independent mutation events in the same site as a single mutation event. This has been previously shown by the 7~8% of doubleton CpG>T shared between distant populations in the same species (**Supplementary Tables 21,22**). Previous studies have suggested that the mutation rate of CpG>T transitions is identical in the great ape lineage [66], and a recent study of de novo mutations in NHGA trios have shown CpG>T represent 17~32% of all mutations in chimpanzee, 14% in gorilla, and 19% in orangutan. This suggests that there are no significant biases in CpG>T mutation rates between human and NHGAs, while there are important differences in the estimates of individual studies[67] or even families[64], while still being similar in all species[66]. In addition to this variation, and the inability of our method to detect recurrent mutations, our choice of analyzing species-exclusive SNV combined with the choice of a human dataset with big sample size (we select species-exclusive SNV in NHGA species comparing against the human dataset 1kGP, with 50x larger sample size than SGDP50) causes additional biases to all estimates of enrichment in CpG>T transitions. Because all of this, we will ignore all the enrichment results obtained in xCG>T trinucleotides, as we will deem them not conclusive.

Correlation with mutation signatures

We downloaded the catalog of COSMIC[68] mutation signatures (v3 May 2019) from https://cancer.sanger.ac.uk/cosmic/signatures/SBS/. We correlated all datasets with Signatures SBS1 and SBS5. These signatures have been described by previous studies to reconstruct the mutation pattern of de novo mutations in human[64,69]. We observe very high correlations of all datasets with signature SBS5, with much lower correlations with SBS1 as expected (SBS1 has few important components) (**Supplementary Table 26**). We combined SBS1 and SBS5 in a single signature, applying a correcting factor to each signature equivalent to the relative weight of CpG mutations (SBS1) or non-CpG mutations (SBS5). This resulted in even stronger correlations with the combined signature. It is worth noting that previous studies[64] used a similar approach but used much higher weights for the CpG mutations (25% in their study, 10% in ours). This difference is probably caused by the recurrent nature of CpG>T transitions. These mutations can be seen as recurrent mutations when using population data, while studies of de novo mutation can observe them in each individual sample.

Species-exclusive variants

We analyzed all trinucleotides in the human, chimpanzee, and gorilla datasets, and determined if each mutation is species-exclusive or it is shared between two or more datasets. The majority of shared variants are CpG>T transitions, as well as a smaller number of T>C transitions. An important caveat of this analysis is that the choice of human dataset will have a very big impact. The 1kGP human dataset has 2,504 samples. It has 1,516,059 ACG>T mutations of which 1,125,648 are species-exclusive, and the rest are shared with chimpanzee, gorilla, or both. The human dataset SGDP50 has 50 samples, 362,142 ACG>T mutations of which 264,804 are species-exclusive. The chimpanzee dataset, when compared with the gorilla and 1kGP human dataset,

has 559,482 species-exclusive ACG>T variants, but, when compared with the SGDP50 human dataset, it has 771,341 species-exclusive ACG>T variants. Gorilla has 184,092 ACG>T when compared with 1kGP, and 254,224 ACG>T when compared with SGDP50.

On one hand, this shows that the choice of human dataset will bias the results of the analysis of CpG>T transitions in other datasets. But, on the other hand, this suggests that these shared CpG>T variants are repeated variants and not shared variants. These variants are at low frequency (<1%) in the human population, as they are not captured by the SGDP50 dataset. Only CpG>T trinucleotides are strongly affected by the choice of dataset.

Conservation of the mutation spectra in primates

We obtained population data from 3 species: the Vervet monkey[70] , Mouse[71], and Pig[72]. We calculated the mutation spectra of each species' population mapped to their own species' reference genome. The mutation spectrum of Vervet monkeys is highly correlated with both signatures SBS1, SBS5, their combination, and with human, chimpanzee and gorilla germlines. Mouse and pig show poorer correlations (**Supplementary Tables 26,27**). This suggests that the mutation spectra are conserved in the primate lineage, and much more so within the great ape lineage.

Whole-Genome enrichment of trinucleotides

We calculated if there is a significant enrichment of certain trinucleotides in human when compared with other NHGA species. To do this, we used the modified chi-square method described in Harris, eLife 2017[73] and set a p-value threshold of 1e-5 to determine its significance. We calculated also the fold-enrichment of each trinucleotide.

The choice of the human dataset, as previously described, has an effect in CpG>T transitions. When comparing species-exclusive trinucleotides in human and chimpanzee, two CpG>T transitions (ACG>T, and TCG>T) show non-coherent fold enrichments when using either the 1kGP or the SGDP50 human datasets (more enriched in human when using 1kGP, more enriched in chimpanzee when using SGDP50). These were the only trinucleotide types affected by the dataset choice. We thus reported as significant only those trinucleotides that showed the same direction of enrichment in both the 1kGP and SGDP50 datasets, reporting the 1kGP fold-enrichment value (**Figure 4a**). Pooling species-exclusive and shared variants, or using only non-singleton variants showed no change in the general enrichment distribution (data not shown).

The top 10% most differently enriched trinucleotides in each of the three comparisons (human – chimpanzee, human – gorilla, chimpanzee – gorilla) were compared against the list of mutation signatures where that trinucleotide appeared at ≥ 5% frequency. Only ATT>A and TTA>A appeared associated with a signature at high frequency (SBS34, 31.6% and 27.2% respectively), although this signature had no associated aetiology yet. The rest of the trinucleotides were associated with signatures in which the trinucleotide has very low frequency or the signature is potentially an artifact (**Supplementary Table 28**).

Our previous analyses have suggested that certain non-CpG mutations in GC-rich windows might have higher mutation density in human than in NHGAs (**Figure 3ef, Supplementary Figure 3**). We hypothesized that this might be detected at trinucleotide level. Thus, we built a linear regression model comparing the log10 fold-enrichment value as a function of the number of C and G in the trinucleotide's 3bp sequence (triplet). This linear model shows that the number of C/G bases in the sequence has a significant effect in the trinucleotide fold enrichment in the human –

chimpanzee comparison (p-value=6.88e-5) (**Supplementary Figure 4**). This significant association holds when including or removing xCG trinucleotides (p-value=3.97e-9) or even when removing TTA>A, the most enriched trinucleotide in chimpanzee (data not shown). This effect is non-significant in human – gorilla comparisons, but significant in chimpanzee – gorilla comparisons. This suggests that GC-rich trinucleotides are more enriched in human than in chimpanzee, but this effect is negligible when comparing human and gorilla. AT-rich trinucleotides are more enriched in chimpanzee than in gorilla.

Distribution of trinucleotides across the genome (trinucleotide-difference test)

We analyzed the distribution across the genome of trinucleotide variants. This allow us to detect if the human-NHGA-tumor diagonal pattern observed using all variants could be observed when using only a given trinucleotide. To do this, we selected 4,920 1Mbp windows where at least 50% of the window passed both the mappabiliy+cnv+callability filter and the reference genome of human, chimpanzee, and gorilla had the same sequence. We counted the number of mutations of each of the 96 trinucleotides in each window, and divided it by the fraction of the window sequence that has that trinucleotide's corresponding 3-bp reference sequence. This gives a measure of mutations / 1Mbp for each trinucleotide mutation and reference, regardless of sequence content. We calculated the standardized mutation distribution in each of the human, chimpanzee, gorilla, and tumor datasets, and also the ranked distribution.

For each window, we created two distributions: in the first one, we subtracted the ranking in the human and tumor datasets. In the second one, we subtracted the ranking in the NHGA (chimpanzee and gorilla separately) and tumor datasets. These human-tumor and chimpanzee-tumor distributions resemble a normal distribution with

mean 0. The standard deviation of these distributions is a magnitude of how similar or different are the tumor and human/NHGA datasets (data not shown). We calculated the two-sided Kolmogorov-Smirnov test (R function ks.test) of both human-tumor and chimpanzee-tumor:

$$KS\ test\ (\ (Rank\ Human - Rank\ Tumor)\ , (Rank\ NHGA - Rank\ Tumor)\ )$$

If human and chimpanzee have a similar relationship with tumor (being either both human and NHGAs similar to tumor, or equally different), both the human-tumor and the chimpanzee-tumor distributions would be similar according to the Kolmogorov-Smirnov test. Otherwise, The KS test will mark both distributions as significantly different. The difference between the standard deviations in both distributions can be used as a magnitude of the effect of how different both tumor-subtracted distributions are (**Figure 4a**).

In addition to the KS-test p-value, we calculated the partial correlation between human – tumor, and chimpanzee – tumor using the R package ppcor. The partial correlation reports the correlation of a given trinucleotide between human and tumor, once the correlation between human and chimpanzee, and chimpanzee and tumor has been factored out. The combination of the KS-test p-value and the partial correlations show good discriminatory power (**Supplementary Figure 4**). All trinucleotides deemed to be significantly different show a strong clustering, with much stronger partial correlations between NHGAs and tumor than between human and tumor.

We did not take into account the SGDP50 human dataset for this trinucleotide-difference analysis because it had a smaller number of SNV than 1kGP. This smaller number of SNV, once divided into 4,920 windows, caused a lot of noise and spurious correlations. Nonetheless, the significant results of ACG>T and TCG>T in

Supplementary Figure 4, again, are probably caused by the effect of different sample size in species-exclusive SNV.

We also modeled the effect that the number of C/G basepairs of the trinucleotide has in the difference of standard deviations results of the trinucleotide-difference test (**Supplementary Figure 4**). We observe no significant association in human-chimpanzee comparisons, and extremely weak correlations in human-gorilla (p-value=0.036) and chimpanzee-gorilla (p-value=0.008) comparisons (data not shown).

Effect of cancer mutational signatures in individual tumor types (signature-difference test)

We ran the trinucleotide-difference test using each individual tumor type instead of pooling all tumor types together. This shows almost none new significant association between individual tumor types and NHGAs that were not detected using all tumors combined. However, some individual tumor types tended to recapitulate the results of using all tumors (**Supplementary Data 1**).

Each individual tumor type is affected in different proportions by the different cancer mutation signatures. We tested if the results of the trinucleotide-difference test in individual tumor types were associated with specific mutation signatures. We downloaded mutation load data for each signature and each tumor type from the PCAWG project[20], and calculated the number of mutations/Mbp per sample associated to each mutation signature in each individual tumor type. For each cancer signature (65 signatures) and trinucleotide (96 trinucleotides) combination, we built a linear regression where the trinucleotide-difference test effect size (the difference between the distributions' standard deviations) of the trinucleotide in each tumor type was dependent on the average mutation load of the signature in that tumor type. We

obtained the p-value associated with the mutation load in this linear model, and run a chi-squared test counting the number of trinucleotides significant (p-value <0.05) in both the trinucleotide-difference test and the linear model. Only two signatures had significant p-value after multiple correction (p-value <1e-4): Signatures SBS5 and SBS40 (**Figure 4b**). Both are flat signatures affecting multiple mutation types. They affect a high number of samples in most if not all tumor types, and have been associated with the age of the patient sample[20,68]. Signature SBS5 has been associated previously with healthy somatic cell mutation accumulation and de novo germline mutations[64,74,75]. Notoriously, signature SBS1 is also associated with the sample's age, but it is not found significant, mostly because signature SBS1 is composed mainly of CpG>T transitions and we detect a similar distribution of those in humans and NHGA in the trinucleotide-difference test.

We repeated this signature-difference test using more complex linear regression models using additional variables: the total number of mutations associated with a signature in all samples in the tumor type, the total number of mutations in the tumor type, and the number of samples in the tumor type. Combinations of these variables with the mutation load of a signature were not informative nor significant in the linear regressions, as these variables are not independent (the total number of signature mutations in a tumor type is dependent on the signature's mutation load and the number of samples; the total number of mutations is dependent on the number of samples and constant across signatures, etc.). Using only the signature's mutation load was the best predictor of the result of the trinucleotide-difference test across tumor types.

Supplementary Tables:

Supplementary Table 1:

Chimpanzee partial correlations. Partial correlations (Pearson's R) comparing 1kGP-chimpanzee-tumor.

	1kGP	Chimpanzee	Tumor
**1kGP**	-	-	-
**Chimpanzee**	0.67	-	-
**Tumor**	-0.3	0.59	-

Supplementary Table 2:

Gorilla partial correlations. Partial Correlations (Pearson's R) comparing 1kGP-gorilla-tumor.

	1kGP	Gorilla	Tumor
**1kGP**	-	-	-
**Gorilla**	0.54	-	-
**Tumor**	-0.21	0.59	-

Supplementary Table 3:

Window size and fraction passing filters. Correlations (Pearson's R) using different window sizes with a minimum window fraction of 50%, or 1Mbp windows with different thresholds for the minimum fraction passing our filtering.

	Variable Window Size passing 50% filter				1Mbp Window Size			
	**10 Mbp**	**1 Mbp**	**100 kbp**	**10 kbp**	**0.5**	**0.6**	**0.7**	**0.8**
**Number of windows**	496	5,040	49,732	476,343	5,040	4,814	4,397	3,037
**Human - Chimpanzee**	0.69	0.65	0.59	0.44	0.65	0.66	0.67	0.68
**Human - Gorilla**	0.54	0.53	0.49	0.36	0.53	0.54	0.55	0.54
**Chimpanzee - Gorilla**	0.86	0.84	0.71	0.48	0.84	0.85	0.84	0.81
**Human - Tumor**	0.05	0.16	0.19	0.19	0.16	0.18	0.18	0.15
**Chimpanzee - Tumor**	0.47	0.55	0.5	0.37	0.55	0.55	0.54	0.49
**Gorilla - Tumor**	0.53	0.58	0.5	0.37	0.58	0.58	0.57	0.52

Supplementary Table 4:

PanTro5 reference. Correlations (Pearson's R) between datasets mapping data to the chimpanzee panTro5 reference instead of the human hg19 reference.

	SGDP50	Chimpanzee 50	Tumor
**SGDP50**	-	-	-
**Chimpanzee 50**	0.77	-	-
**Tumor**	0.25	0.52	-

Supplementary Table 5:

NHGA subspecies and additional datasets. Pairwise correlations (Pearson's R) between diverse datasets using all sites. 1kGP: 1000 Genomes Project 2,504 samples; 1kGP 69: 1000 Genomes Project 69 samples; 1kGP 43: 1000 Genomes Project 43 samples; ICGC: release of 1,823 germline samples from the PanCancer Analysis of Whole Genomes; SGDP279: release of 279 samples from Simons Genome Diversity Project; SGDP50: our calling of 50 human samples from SGDP; Africa 26: Subset of SGDP50 with 26 samples of African origin; Not Africa 24: Subset of SGDP50 with 24 samples of non-African origin. Chimpanzee: our calling of 69 chimpanzee; pp: *Pan paniscus* (bonobo) 10 samples; ptv: *Pan troglodytes verus* 12 samples; pte: *Pan troglodytes ellioti* 10 samples; pts: *Pan troglodytes schweinfurthii* 19 samples; ptt: *Pan troglodytes troglodytes* 18 samples; ptt 5: subset of 5 samples of ptt. Gorilla: our calling of 43 gorilla samples; gbb: *Gorilla beringei beringei* 7 samples; gbg: *Gorilla beringei graueri* 8 samples; ggg: *Gorilla gorilla gorilla* 27 samples; ggg 5: subset of 5 samples of ggg. Tumor: release of 2,583 human tumor samples from PCAWG. Window ratio: fraction of the window passing filters; GC-content of the window; CpG dinucleotide content of the window. NS: non-significant (Correlation test p-value > 0.05).

	1kGP	1kGP 69	1kGP 43	ICGC	SGDP279	SGDP50	African 26	Non-African 24	Chimpanzee	pp	ptv	pte	pts	ptt	ptt 5	Gorilla	gbb	gbg	ggg	ggg 5	Tumor	Window ratio	GC-content	CpG
**1kGP**	-	-	-	-	-	-	-	-	-	-	-	-	-	-	-	-	-	-	-	-	-	-	-	-
**1kGP 69**	0.9	-	-	-	-	-	-	-	-	-	-	-	-	-	-	-	-	-	-	-	-	-	-	-
**1kGP 43**	0.88	0.99	-	-	-	-	-	-	-	-	-	-	-	-	-	-	-	-	-	-	-	-	-	-
**ICGC**	0.99	0.91	0.89	-	-	-	-	-	-	-	-	-	-	-	-	-	-	-	-	-	-	-	-	-
**SGDP279**	0.94	0.97	0.96	0.95	-	-	-	-	-	-	-	-	-	-	-	-	-	-	-	-	-	-	-	-
**SGDP50**	0.91	0.97	0.96	0.91	0.97	-	-	-	-	-	-	-	-	-	-	-	-	-	-	-	-	-	-	-
**African 26**	0.89	0.96	0.96	0.89	0.95	0.99	-	-	-	-	-	-	-	-	-	-	-	-	-	-	-	-	-	-
**Non-African 24**	0.79	0.92	0.92	0.81	0.89	0.92	0.89	-	-	-	-	-	-	-	-	-	-	-	-	-	-	-	-	-
**Chimpanzee**	0.65	0.69	0.69	0.64	0.7	0.74	0.74	0.65	-	-	-	-	-	-	-	-	-	-	-	-	-	-	-	-
**pp**	0.48	0.57	0.57	0.49	0.56	0.59	0.59	0.55	0.76	-	-	-	-	-	-	-	-	-	-	-	-	-	-	-
**ptv**	0.45	0.5	0.5	0.46	0.5	0.51	0.51	0.48	0.6	0.46	-	-	-	-	-	-	-	-	-	-	-	-	-	-
**pte**	0.51	0.61	0.61	0.52	0.6	0.63	0.63	0.59	0.82	0.61	0.53	-	-	-	-	-	-	-	-	-	-	-	-	-
**pts**	0.57	0.66	0.66	0.58	0.65	0.69	0.7	0.63	0.92	0.67	p	0.81	-	-	-	-	-	-	-	-	-	-	-	-
**ptt**	0.6	0.66	0.66	0.6	0.66	0.71	0.71	0.63	0.96	0.69	0.58	0.82	0.92	-	-	-	-	-	-	-	-	-	-	-
**ptt 5**	0.58	0.66	0.66	0.58	0.65	0.69	0.7	0.63	0.93	0.67	0.57	0.81	0.91	0.96	-	-	-	-	-	-	-	-	-	-
**Gorilla**	0.53	0.64	0.64	0.53	0.63	0.68	0.68	0.62	0.84	0.66	0.52	0.73	0.8	0.82	0.8	-	-	-	-	-	-	-	-	-
**gbb**	0.32	0.4	0.41	0.32	0.39	0.42	0.43	0.4	0.53	0.43	0.32	0.47	0.5	0.52	0.5	0.64	-	-	-	-	-	-	-	-
**gbg**	0.37	0.45	0.46	0.37	0.44	0.47	0.47	0.45	0.55	0.42	0.39	0.5	0.54	0.54	0.53	0.67	0.5	-	-	-	-	-	-	-
**ggg**	0.51	0.62	0.62	0.51	0.61	0.66	0.67	0.61	0.83	0.66	0.51	0.72	0.79	0.82	0.79	0.99	0.59	0.62	-	-	-	-	-	-
**ggg 5**	0.46	0.58	0.58	0.46	0.56	0.62	0.63	0.57	0.81	0.64	0.49	0.7	0.77	0.79	0.77	0.96	0.58	0.6	0.97	-	-	-	-	-
**Tumor**	0.16	0.22	0.22	0.18	0.25	0.26	0.27	0.22	0.55	0.42	0.32	0.46	0.5	0.52	0.5	0.58	0.38	0.37	0.58	0.59	-	-	-	-
**Window ratio**	NS	0.03	0.03	NS	NS	0.08	0.08	0.06	0.32	0.22	0.12	0.25	0.29	0.31	0.28	0.31	0.18	0.18	0.32	0.34	0.36	-	-	-
**GC-content**	0.25	0.15	0.14	0.23	0.15	0.1	0.08	0.08	-0.24	-0.21	-0.13	-0.19	-0.22	-0.25	-0.23	-0.24	-0.18	-0.15	-0.25	-0.3	-0.59	-0.42	-	-
**CpG**	0.37	0.25	0.24	0.36	0.28	0.2	0.19	0.17	-0.12	-0.12	-0.04	-0.1	-0.12	-0.13	-0.12	-0.15	-0.12	-0.1	-0.16	-0.23	-0.49	-0.5	0.91	-

Supplementary Table 6:

Orangutan. Pairwise correlations (Pearson's R) between datasets using orangutan samples.

	1kGP	Chimpanzee	Gorilla	Orangutan (all)	*P. abelii*	*P. pygmaeus*	Tumor
**1kGP**	-	-	-	-	-	-	-
**Chimpanzee**	0.66	-	-	-	-	-	-
**Gorilla**	0.55	0.84	-	-	-	-	-
**Orangutan (all)**	0.43	0.69	0.76	-	-	-	-
Pongo abelii	0.41	0.66	0.73	0.97	-	-	-
Pongo pygmaeus	0.37	0.63	0.71	0.91	0.81	-	-
**Tumor**	0.18	0.55	0.58	0.55	0.52	0.54	-

Supplementary Table 7:

Allele frequency. Correlation (Pearson's R) between different pairs of species using only variants of a certain frequency. AF: Allele Frequency.

	1kGP - Chimpanzee	1kGP - Gorilla	Chimpanzee - Gorilla	1kGP - Tumor	Chimpanzee - Tumor	Gorilla - Tumor
**All segregating SNV**	0.65	0.53	0.84	0.16	0.55	0.58
**Singletons**	0.49	0.25	0.53	0.15	0.47	0.43
**Doubletons**	0.38	0.19	0.46	-0.02	0.45	0.39
**Non-singletons**	0.67	0.55	0.83	0.16	0.54	0.59
**AF ≤ 0.05**	0.63	0.57	0.71	0.2	0.53	0.44
**AF > 0.05**	0.65	0.58	0.79	0.2	0.5	0.59
**AF ≤ 0.1**	0.65	0.6	0.78	0.21	0.54	0.51
**AF > 0.1**	0.61	0.56	0.73	0.19	0.47	0.58

Supplementary Table 8:

Allele frequencies within dataset. Correlation (Pearson's R) between variants at different frequencies within each dataset. AF: Allele Frequency. NHGA subspecies are defined in Supplementary Table 5.

	Non-singletons vs Singletons	AF <= 5% vs AF > 5%	AF <= 10% vs AF > 10%
**1kGP**	0.86	0.60	0.60
**1kGP 69**	0.70	0.71	0.73
**1kGP 43**	0.69	0.73	0.75
**SGDP50**	0.72	-	-
**Chimpanzee**	0.78	0.80	0.75
**pp**	0.35	-	-
**ptv**	0.23	-	-
**pte**	0.45	-	-
**pts**	0.61	-	-
**ptt**	0.75	-	-
**Gorilla**	0.57	0.72	0.79
**ggg**	0.61	-	-
**gbb**	-0.08	-	-
**gbg**	0.02	-	-

Supplementary Table 9:

Species-exclusive SNV. Correlation (Pearson's R) between pairs of species/datasets when using only variants exclusively found in that species.

	SNV exclusive of a species (not applied to tumor)
**1kGP exclusive vs Chimpanzee exclusive**	0.55
**1kGP exclusive vs Gorilla exclusive**	0.43
**1kGP exclusive vs Tumor**	0.16
**Chimpanzee exclusive vs Tumor**	0.60
**Gorilla exclusive vs Tumor**	0.62

Supplementary Table 10:

Species-exclusive vs shared SNV. Correlation (Pearson's R) between variants exclusive in one species and variants shared between pairs of human-NHGA species. Exclusivity/sharedness does not apply to the tumor dataset.

	Shared
1kGP exclusive vs shared 1kGP-Chimpanzee	0.64
Chimpanzee exclusive vs shared 1kGP-Chimpanzee	0.42
Tumor vs shared 1kGP-Chimpanzee	-0.02
1kGP exclusive vs shared 1kGP-Gorilla	0.59
Gorilla exclusive vs shared 1kGP-Gorilla	0.60
Tumor vs shared 1kGP-Gorilla	0.13

Supplementary Table 11:

Divergence. Correlation (Pearson's R) between human-chimpanzee divergence or human-gorilla divergence and all segregating sites in each dataset.

	Human-Chimpanzee divergence	Human-Gorilla divergence
**1kGP**	0.29	0.24
**SGDP50**	0.26	0.22
**Chimpanzee**	0.20	0.19
**Chimpanzee 50 samples**	0.20	0.20
**Gorilla**	0.24	0.13
**Tumor**	0.18	0.10

Supplementary Table 12:

InDels. Correlation (Pearson's R) between SNV mutation density and InDel mutation density in the different datasets, and between InDel mutation density in pairs of datasets.

	InDels 1kGP	InDels SGDP50	InDels Chimpanzee	InDels Gorilla	InDels Tumor
**SNV 1kGP**	0.49	0.34	0.35	0.31	-0.02
**SNV SGDP50**	0.6	0.48	0.39	0.43	0.07
**SNV Chimpanzee**	0.43	0.26	0.49	0.53	0.39
**SNV Gorilla**	0.42	0.28	0.38	0.66	0.41
**SNV Tumor**	0.18	0.04	0.17	0.41	0.9
**InDels 1kGP**	-	-	-	-	-
**InDels SGDP50**	0.83	-	-	-	-
**InDels Chimpanzee**	0.69	0.64	-	-	-
**InDels Gorilla**	0.64	0.57	0.69	-	-
**InDels Tumor**	0.16	0.05	0.21	0.42	-

Supplementary Table 13:

Archaic samples. Correlations (Pearson's R) between datasets using the filtering derived from archaic samples.

	1kGP	Archaic	Chimpanzee	Gorilla	Tumor
**1kGP**	-	-	-	-	-
**Archaic**	0.67	-	-	-	-
**Chimpanzee**	0.66	0.58	-	-	-
**Gorilla**	0.55	0.55	0.84	-	-
**Tumor**	0.18	0.22	0.53	0.56	-

Supplementary Table 14:

Tumor types. Pearson's correlation R between human, chimpanzee, or gorilla standardized mutation density in 1Mbp windows, and the mutation density of tumors of different histologies, and tumor subsets by sex and age. Tumor histologies are defined as in the original PCAWG dataset. The last column denotes the two-sided Mann-Whitney U test of measuring the standardized mutation density in tumor grouping windows by being above or below the diagonal when comparing 1kGP and chimpanzee ranked mutation densities.

Tumor type	# samples	# SNV	Pearson's R human	Pearson's R chimpanzee	Pearson's R gorilla	Mann-Whitney U test p-value (two-sided)
All	2,583	34,911,808	0.16	0.55	0.58	3.7E-216
Skin-Melanoma	107	9,601,997	0.16	0.46	0.49	1.8E-118
ColoRect-AdenoCA	52	7,281,777	0.08	0.5	0.52	1.5E-294
Liver-HCC	315	3,076,372	0.1	0.49	0.51	1.7E-221
Eso-AdenoCa	97	2,136,133	0.17	0.51	0.53	2.5E-186
Lung-SCC	47	1,616,150	0.09	0.46	0.5	1.1E-200
Stomach-AdenoCA	68	1,263,860	0.18	0.52	0.53	1.1E-180
Panc-AdenoCA	232	1,183,031	0.21	0.49	0.52	6.5E-123
Breast-AdenoCa	195	1,063,688	0.17	0.39	0.4	5E-86
Lung-AdenoCA	37	1,009,893	0.18	0.52	0.56	4.3E-156
Lymph-BNHL	105	985,991	0.13	0.41	0.42	2.2E-215
Ovary-AdenoCA	110	758,465	0.16	0.45	0.47	4.5E-107
Uterus-AdenoCA	44	736,102	0.13	0.45	0.47	1.9E-191
Kidney-RCC	143	733,510	0.14	0.43	0.46	6.2E-97
Head-SCC	56	681,568	0.11	0.47	0.5	1.6E-195
Prost-AdenoCA	199	465,385	0.17	0.46	0.47	2.8E-132
Bladder-TCC	23	391,265	0.14	0.39	0.4	5.5E-80
CNS-GBM	39	377,586	0.2	0.38	0.42	3E-15
Biliary-AdenoCA	33	373,854	0.18	0.49	0.5	3.1E-150
Panc-Endocrine	81	192,608	0.19	0.47	0.49	7.6E-95
Lymph-CLL	90	171,011	0.13	0.45	0.46	1.7E-174
CNS-Medullo	141	150,130	0.15	0.42	0.44	1.9E-81
Bone-Leiomyo	34	140,410	0.13	0.34	0.38	5E-102
Bone-Osteosarc	41	117,874	0.2	0.48	0.51	8.9E-104
Cervix-SCC	18	84,315	0.14	0.35	0.36	7.2E-69
Breast-LobularCa	13	71,875	0.08	0.2	0.2	1.8E-19
Kidney-ChRCC	43	60,390	0.28	0.45	0.44	3.4E-51
Thy-AdenoCA	47	46,867	0.17	0.38	0.43	1.9E-54
CNS-Oligo	18	34,853	0.12	0.38	0.41	1.3E-66
Lymph-NOS	2	21,545	0.1	0.38	0.4	5.7E-162
Myeloid-MPN	24	18,753	0.2	0.36	0.39	1E-30
Bone-Epith	11	17,772	0.1	0.35	0.37	5E-84
CNS-PiloAstro	89	15,158	0.06	0.3	0.32	8.3E-66
Myeloid-AML	12	13,546	0.15	0.33	0.36	2.7E-36
Cervix-AdenoCA	2	5,998	0.11	0.24	0.24	2.3E-21
Bone-Cart	9	5,659	0.08	0.28	0.31	1E-45
Breast-DCIS	3	4,443	0.09	0.18	0.18	3.3E-15
Testicular Germ Cell	8	2,710	0.03	0.06	0.06	0.84
Myeloid-MDS	1	403	0.03	0.05	0.04	0.09
Male	1,420	24,377,637	0.15	0.54	0.58	6.40E-226
Male no sex-specific	1,220	23,910,412	0.15	0.54	0.58	2.60E-226
Female	1,160	10,515,068	0.19	0.55	0.58	8.40E-186
Female no sex-specific	776	7,792,022	0.19	0.56	0.59	1.10E-186
< 30 years	387	1,909,188	0.16	0.49	0.52	1.00E-158
30-59 years	867	13,220,016	0.14	0.54	0.57	1.40E-241
≥ 60 years	1,328	19,782,596	0.17	0.55	0.58	9.90E-202

Supplementary Table 15:

Tumors with >1M SNV. Pairwise correlations (Pearson's R) between the most mutated tumor types (>1M SNVs in the dataset). The Breast-Adenocarcinoma dataset was split into two groups corresponding to samples presenting or not signals of mutational signature SBS3. Tumor histologies defined as in the original PCAWG dataset.

	All Tumors	Skin-Melanoma	ColoRect-AdenoCA	Liver-HCC	Eso-AdenoCa	Lung-SCC	Stomach-AdenoCA	Panc-AdenoCA	Breast-AdenoCa	Lung-AdenoCA	Breast-AdenoCa - SBS3	Breast-AdenoCa - NO SBS3
All Tumors	-	-	-	-	-	-	-	-	-	-	-	-
Skin-Melanoma	0.88	-	-	-	-	-	-	-	-	-	-	-
ColoRect-AdenoCA	0.90	0.66	-	-	-	-	-	-	-	-	-	-
Liver-HCC	0.87	0.65	0.83	-	-	-	-	-	-	-	-	-
Eso-AdenoCa	0.91	0.69	0.87	0.81	-	-	-	-	-	-	-	-
Lung-SCC	0.88	0.67	0.79	0.78	0.82	-	-	-	-	-	-	-
Stomach-AdenoCA	0.92	0.69	0.89	0.82	0.97	0.82	-	-	-	-	-	-
Panc-AdenoCA	0.87	0.63	0.81	0.79	0.93	0.8	0.91	-	-	-	-	-
Breast-AdenoCa	0.69	0.57	0.54	0.6	0.64	0.71	0.66	0.65	-	-	-	-
Lung-AdenoCA	0.88	0.7	0.78	0.78	0.83	0.86	0.83	0.83	0.69	-	-	-
Breast-AdenoCa - SBS3	0.65	0.54	0.5	0.56	0.59	0.68	0.61	0.6	0.93	0.64	-	-
Breast-AdenoCa - NO SBS3	0.65	0.52	0.51	0.56	0.61	0.64	0.63	0.62	0.94	0.65	0.75	-

Supplementary Table 16:

Source of the different genomic features used.

Feature	Source	Type
GC-content	Calculated from the fraction of base pairs in a window passing our filters and being G or C in the human reference hg19	Overlap
CpG	Calculated from the fraction of base pairs in a window passing our filters and being consecutive CpG in the human reference hg19	Overlap
CpG islands	UCSC Genome Browser track: cpgIslandExt	Overlap
CpG methylation sites	UCSC Genome Browser track: CpG Methylation by Methyl 450K Bead Arrays from ENCODE/HAIB. Signal > 200	Overlap
CpG hypermethylated in human/chimpanzee/gorilla	From Hernando-Herraez, I., Plos Genetics 2013. CpG sites marked as differently methylated in a species with no differences in other genera, and higher β-value in this species than in Orangutan	Overlap
CpG hypomethylated in human/chimpanzee/gorilla	From Hernando-Herraez, I., Plos Genetics 2013. CpG sites marked as differently methylated in a species with no differences in other genera, and lower β-value in this species than in Orangutan	Overlap
Genes	Protein-Coding Genes coordinates from ENSEMBL Genes 95 for human GRCh37.p13	Overlap
Exons	Exon coordinates of Protein-Coding Genes from ENSEMBL Genes 95 for human GRCh37.p13	Overlap
Recombination in Human (Male/Female/Average)	UCSC Genome Browser track: Recombination Rate from deCODE	Intensity
Recombination in Human PRDM9 DBS hotspots	From Pratto, F., Science 2014. Maps of Double Strand Breaks initiatied by PRDM9 binding sites.	Overlap
Recombination in Chimpanzee (Auton)	From Auton, A., Science 2012. Hotspots of recombination. Using *Pan troglodytes verus*	Overlap
Recombination in Chimpanzee (Stevison)	From Stevison, L., Mol Biol Evol 2015. Measures of rho/kb across the genome in Chimpanzee samples (*Pan troglodytes ellioti*)	Intensity
Recombination in Bonobo (Stevison)	From Stevison, L., Mol Biol Evol 2015. Measures of rho/kb across the genome in Bonobo samples	Intensity
Recombination in Gorilla (Stevison)	From Stevison, L., Mol Biol Evol 2015. Measures of rho/kb across the genome in Gorilla samples (*Gorilla gorilla gorilla*)	Intensity
Mappability of 24-mers	UCSC Genome Browser track: Mappability or Uniqueness of Reference Genome from ENCODE.	Intensity
Nucleosome Positioning	From Schones, D.E., Cell 2008. Downloaded from UCSC Genome Browser track: wgEncodeSydhNsomeGm12878Sig from ENCODE (Stanford, Snyder lab) using GM12878 cell line	Intensity
Methylation histone marks, H2AZ, CTCF and PolII binding	From Barski, A., Cell 2007. Measures of read-depth in CD4+ T cells	Intensity
Acetylation Histone Marks	From Wang, Z., Nature Genetics 2008. Measures of read-depth in CD4+ T cells	Intensity
Hi-C	From Lieberman-Aiden, E., Science 2009.	Intensity
Replication time	From Hansen, R.S., Proc. Natl Acad. Sci. 2010. Early-to-late ratio.	Intensity
Conservation	UCSC Genome Browser track: Conservation using 100 vertebrates - Vertebrate Multiz Alignment & Conservation (100 Species) - phyloP100wayAll	Intensity
Human ChromHMM chromatin states	From UCSC Genome Browser track: Broad ChromHMM - Chromatin State Segmentation by HMM from ENCODE/Broad in the GM12878 cell line.	Overlap

Supplementary Table 17:

Subspecies non-CpG sites. Pairwise correlations (Pearson's R) between diverse datasets using only non-CpG sites. Subspecies defined in Supplementary Table 5.

	1kGP	1kGP 69	SGDP50	Human de novo	Chimpanzee	pp	ptv	pte	pts	ptt	ptt 5	Gorilla	gbb	gbg	ggg	ggg 5	Tumor
**1kGP**	-	-	-	-	-	-	-	-	-	-	-	-	-	-	-	-	-
**1kGP 69**	0.9	-	-	-	-	-	-	-	-	-	-	-	-	-	-	-	-
**SGDP50**	0.92	0.96	-	-	-	-	-	-	-	-	-	-	-	-	-	-	-
**Human de novo**	0.44	0.35	0.36	-	-	-	-	-	-	-	-	-	-	-	-	-	-
**Chimpanzee**	0.7	0.68	0.74	0.27	-	-	-	-	-	-	-	-	-	-	-	-	-
**pp**	0.56	0.59	0.62	0.21	0.8	-	-	-	-	-	-	-	-	-	-	-	-
**ptv**	0.52	0.53	0.55	0.2	0.64	0.52	-	-	-	-	-	-	-	-	-	-	-
**pte**	0.58	0.63	0.65	0.22	0.84	0.66	0.58	-	-	-	-	-	-	-	-	-	-
**pts**	0.64	0.67	0.71	0.24	0.94	0.72	0.62	0.83	-	-	-	-	-	-	-	-	-
**ptt**	0.66	0.67	0.72	0.25	0.97	0.74	0.62	0.84	0.94	-	-	-	-	-	-	-	-
**ptt 5**	0.64	0.67	0.71	0.24	0.94	0.72	0.62	0.84	0.92	0.97	-	-	-	-	-	-	-
**Gorilla**	0.59	0.64	0.69	0.21	0.86	0.7	0.57	0.76	0.82	0.84	0.82	-	-	-	-	-	-
**gbb**	0.38	0.43	0.45	0.13	0.56	0.47	0.36	0.51	0.53	0.54	0.53	0.66	-	-	-	-	-
**gbg**	0.42	0.48	0.5	0.15	0.58	0.46	0.43	0.53	0.57	0.57	0.56	0.69	0.52	-	-	-	-
**ggg**	0.58	0.63	0.68	0.21	0.85	0.7	0.56	0.75	0.82	0.84	0.82	0.99	0.61	0.64	-	-	-
**ggg 5**	0.56	0.61	0.66	0.2	0.84	0.69	0.54	0.74	0.8	0.82	0.8	0.97	0.6	0.63	0.98	-	-
**Tumor**	0.38	0.34	0.39	0.17	0.66	0.53	0.41	0.55	0.6	0.62	0.6	0.66	0.43	0.44	0.66	0.66	-

Supplementary Table 18:

Subspecies CpG>T. Pairwise correlations (Pearson's R) between diverse datasets using only CpG>T transitions. Subspecies defined in Supplementary Table 5.

	1kGP	1kGP 69	SGDP50	Human de novo	Chimpanzee	pp	ptv	pte	pts	ptt	ptt 5	Gorilla	gbb	gbg	ggg	ggg 5	Tumor
**1kGP**	-	-	-	-	-	-	-	-	-	-	-	-	-	-	-	-	-
**1kGP 69**	0.97	-	-	-	-	-	-	-	-	-	-	-	-	-	-	-	-
**SGDP50**	0.97	0.99	-	-	-	-	-	-	-	-	-	-	-	-	-	-	-
**Human de novo**	0.63	0.6	0.6	-	-	-	-	-	-	-	-	-	-	-	-	-	-
**Chimpanzee**	0.98	0.95	0.96	0.61	-	-	-	-	-	-	-	-	-	-	-	-	-
**pp**	0.91	0.91	0.91	0.56	0.94	-	-	-	-	-	-	-	-	-	-	-	-
**ptv**	0.85	0.83	0.84	0.54	0.86	0.8	-	-	-	-	-	-	-	-	-	-	-
**pte**	0.91	0.92	0.92	0.57	0.94	0.88	0.82	-	-	-	-	-	-	-	-	-	-
**pts**	0.95	0.94	0.94	0.59	0.97	0.91	0.84	0.94	-	-	-	-	-	-	-	-	-
**ptt**	0.96	0.95	0.95	0.59	0.99	0.92	0.85	0.95	0.98	-	-	-	-	-	-	-	-
**ptt 5**	0.94	0.94	0.94	0.58	0.97	0.91	0.85	0.94	0.97	0.99	-	-	-	-	-	-	-
**Gorilla**	0.93	0.94	0.95	0.57	0.94	0.9	0.82	0.91	0.93	0.94	0.93	-	-	-	-	-	-
**gbb**	0.67	0.71	0.71	0.42	0.69	0.66	0.59	0.69	0.7	0.7	0.69	0.77	-	-	-	-	-
**gbg**	0.76	0.78	0.79	0.47	0.77	0.74	0.69	0.75	0.77	0.77	0.77	0.84	0.7	-	-	-	-
**ggg**	0.91	0.94	0.94	0.56	0.93	0.9	0.81	0.9	0.92	0.93	0.93	1	0.75	0.81	-	-	-
**ggg 5**	0.87	0.9	0.91	0.53	0.89	0.87	0.77	0.87	0.89	0.9	0.89	0.97	0.74	0.8	0.98	-	-
**Tumor**	-0.13	-0.04	-0.05	-0.09	-0.14	-0.09	-0.09	-0.06	-0.08	-0.09	-0.08	-0.01	0.06	0.01	0	0.04	-

Supplementary Table 19:

Orangutan non-CpG. Pairwise correlations (Pearson's R) between diverse datasets when filtered using orangutan samples and using only non-CpG sites.

	1kGP	Chimpanzee	Gorilla	Orangutan	Tumor
**1kGP**	-	-	-	-	-
**Chimpanzee**	0.7	-	-	-	-
**Gorilla**	0.6	0.86	-	-	-
**Orangutan**	0.45	0.71	0.78	-	-
**Tumor**	0.38	0.66	0.66	0.65	-

Supplementary Table 20:

Orangutan CpG>T. Pairwise correlations (Pearson's R) between diverse datasets when filtered using orangutan samples and using only CpG>T transitions.

	1kGP	Chimpanzee	Gorilla	Orangutan	Tumor
**1kGP**	-	-	-	-	-
**Chimpanzee**	0.98	-	-	-	-
**Gorilla**	0.93	0.94	-	-	-
**Orangutan**	0.96	0.96	0.93	-	-
**Tumor**	0.41	0.4	0.49	0.38	-

Supplementary Table 21:

Non-CpG / CpG>T fraction. Fraction of CpG and non-CpG sites compared with the total number variants in the dataset, the non-singleton variants, and the average number of heterozygous sites per sample.

	Fraction of ALL SNV			Fraction of Non-singleton SNV			Fraction of average heterozygous SNV per sample		
	Count All	Non-CpG	CpG	Count All	Non-CpG	CpG	Count All	Non-CpG	CpG
**1kGP**	47,872,324	89.66%	10.34%	24,837,444	88.39%	11.61%	-	-	-
**SGDP50**	10,651,708	89.04%	10.96%	5,845,807	89.06%	10.94%	912,555	89.28%	10.72%
**Chimpanzee**	26,872,437	89.91%	10.09%	18,741,493	89.69%	10.31%	1,408,912	89.67%	10.33%
**Gorilla**	9,813,559	89.22%	10.78%	8,431,675	89.25%	10.75%	1,289,016	89.59%	10.41%
**Tumor**	30,756,260	94.96%	5.04%	-	-	-	-	-	-

Supplementary Table 22:

Fraction of CpG doubletons. Fraction of doubletons at CpG sites shared between samples of different subspecies/populations using all mutations at CpG sites or exclusively CpG>T transitions. NHGA subspecies defined in Supplementary Table 5.

	% of doubletons at CpG sites shared between groups	% of doubletons at CpG>T shared between groups
**SGDP50 - African / non-African**	6.91%	7.10%
**Chimpanzee - pp / ptv / pte / pts /ptt**	11.66%	12.01%
**Chimpanzee - pp / (ptv+pte) / (pts+ptt)**	7.62%	7.89%
**Gorilla - ggg / gbb / gbg**	6.10%	6.13%

Supplementary Table 23:

De novo mutations. Correlation of the standardized mutation distribution between 77,000 human de novo mutations from Carlson 2018 compared with other datasets, using all variants; using only SNV at non-CpG sites; and using only CpG>T transitions. Due to the smaller size of the de novo mutations dataset, multiple correlation methods are reported.

		1kGP	SGDP50	Chimpanzee	Gorilla	Tumor
All SNV	Pearson's R	0.48	0.39	0.24	0.18	0.04
	Poisson Regression R^2	0.21	0.15	0.06	0.03	0
	Negative Binomial Regression R^2	0.2	0.14	0.06	0.03	0
non-CpG sites	Pearson's R	0.44	0.36	0.27	0.22	0.17
	Poisson Regression R^2	0.18	0.13	0.08	0.05	0.03
	Negative Binomial Regression R^2	0.17	0.12	0.08	0.05	0.03
CpG>T	Pearson's R	0.63	0.6	0.61	0.57	-0.09
	Poisson Regression R^2	0.32	0.29	0.3	0.27	0.01
	Negative Binomial Regression R^2	0.3	0.28	0.29	0.25	0.01

Supplementary Table 24:

Within-species correlation of CpG>T vs non-CpG. Correlation (Pearson's R) between CpG>T transitions and mutations at non-CpG sites (correcting by the CpG content of the sequence) between windows of the same species' dataset. NHGA subspecies defined in Supplementary Table 5.

	CpG>T vs non-CpG
**1kGP**	0.39
**1kGP 69**	0.54
**SGDP50**	0.56
**Human de novo**	0.06
**Chimpanzee**	0.67
**pp**	0.72
**ptv**	0.8
**pte**	0.77
**pts**	0.74
**ptt**	0.73
**Gorilla**	0.84
**gbb**	0.93
**gbg**	0.9
**ggg**	0.85
**Orangutan**	0.71
**Tumor**	0.94

Supplementary Table 25:

3-references filter. Fraction of trinucleotides at non-CpG and CpG sites passing the 3-references filter (identical trinucleotide sequence in the hg19, panTro5, and gorGor5 reference) in each dataset.

	non-CpG	CpG
**1kGP**	0.95	0.85
**SGDP50**	0.94	0.84
**Chimpanzee**	0.86	0.81
**Gorilla**	0.7	0.66

Supplementary Table 26:

Signatures SBS1 & SBS5. Correlation (Pearson's R) of the species' mutation spectrum with mutation signatures SBS1, SBS5, and a combination of both signatures weighed by the proportion of CpG>T transitions in the human dataset.

	SBS1	SBS5	10%*SBS1 + 90%SBS5
**1kGP**	0.4	0.92	0.97
**SGDP50**	0.42	0.91	0.97
**Chimpanzee**	0.36	0.92	0.95
**Gorilla**	0.36	0.92	0.95
**panTro5 SGDP50**	0.36	0.93	0.96
**panTro5 Chimpanzee 50**	0.36	0.92	0.95
**Vervet Monkey**	0.52	0.82	0.95
**Mouse**	0.13	0.82	0.74
**Pig**	0.4	0.75	0.83

Supplementary Table 27:

Mutation spectra correlation. Pairwise correlation (Pearson's R) of mutation spectra between species/datasets.

	1kGP	SGDP50	Chimpanzee	Gorilla	panTro5 SGDP50	panTro5 Chimpanzee 50	Vervet Monkey	Mouse	Pig
**1kGP**	-	-	-	-	-	-	-	-	-
**SGDP50**	1	-	-	-	-	-	-	-	-
**Chimpanzee**	0.99	0.99	-	-	-	-	-	-	-
**Gorilla**	0.99	0.99	0.99	-	-	-	-	-	-
**panTro5 SGDP50**	0.99	0.99	0.98	0.99	-	-	-	-	-
**panTro5 Chimpanzee 50**	0.98	0.98	0.99	0.98	0.99	-	-	-	-
**Vervet Monkey**	0.93	0.93	0.93	0.91	0.94	0.95	-	-	-
**Mouse**	0.83	0.82	0.82	0.79	0.77	0.78	0.87	-	-
**Pig**	0.82	0.82	0.82	0.8	0.82	0.83	0.85	0.8	-
**All tumors**	0.39	0.37	0.38	0.36	-	-	0.41	0.58	0.42

Supplementary Table 28:

Enriched trinucleotides presence in signatures. Frequency at which the top 10% most enriched trinucleotides in human-chimpanzee, human-gorilla, and chimpanzee-gorilla comparisons appear in different tumor mutation signatures of the COSMIC catalog.

	ACC>A	ACT>A	CCG>A	GCG>A	TCG>A	ATT>A	TTA>A	ATG>G	CTG>G	GTG>G	TTG>G	Proposed aetiology
**SBS7c**						0.051						UV-light damage
**SBS14**		0.083										Defective DNA mismatch repair (MSI)
**SBS22**							0.072					Aristolochic acid exposure
**SBS24**				0.066								Aflatoxin exposure
**SBS27**							0.053					Possible sequencing artifact
**SBS29**	0.051											Tobacco chewing habit
**SBS34**						0.316	0.272					Unknown
**SBS38**			0.051									UV-light damage
**SBS41**						0.070	0.081					Unknown
**SBS43**										0.241		Possible sequencing artifact
**SBS45**			0.068									Possible sequencing artifact
**SBS47**						0.068	0.156					Possible sequencing artifact
**SBS48**			0.137		0.709							Possible sequencing artifact
**SBS49**			0.515									Possible sequencing artifact
**SBS50**	0.127	0.083										Possible sequencing artifact
**SBS51**										0.076		Possible sequencing artifact
**SBS52**					0.134							Possible sequencing artifact
**SBS53**			0.068	0.051								Possible sequencing artifact
**SBS55**								0.145	0.113	0.299	0.080	Possible sequencing artifact
**SBS60**										0.662		Possible sequencing artifact
**SBS85**						0.070	0.060					Indirect effects of activation-induced cytidine deaminase (AID) induced somatic mutagenesis in lymphoid cells.

Supplementary Figures:

Suppl. Figure 1 Robustness of the results

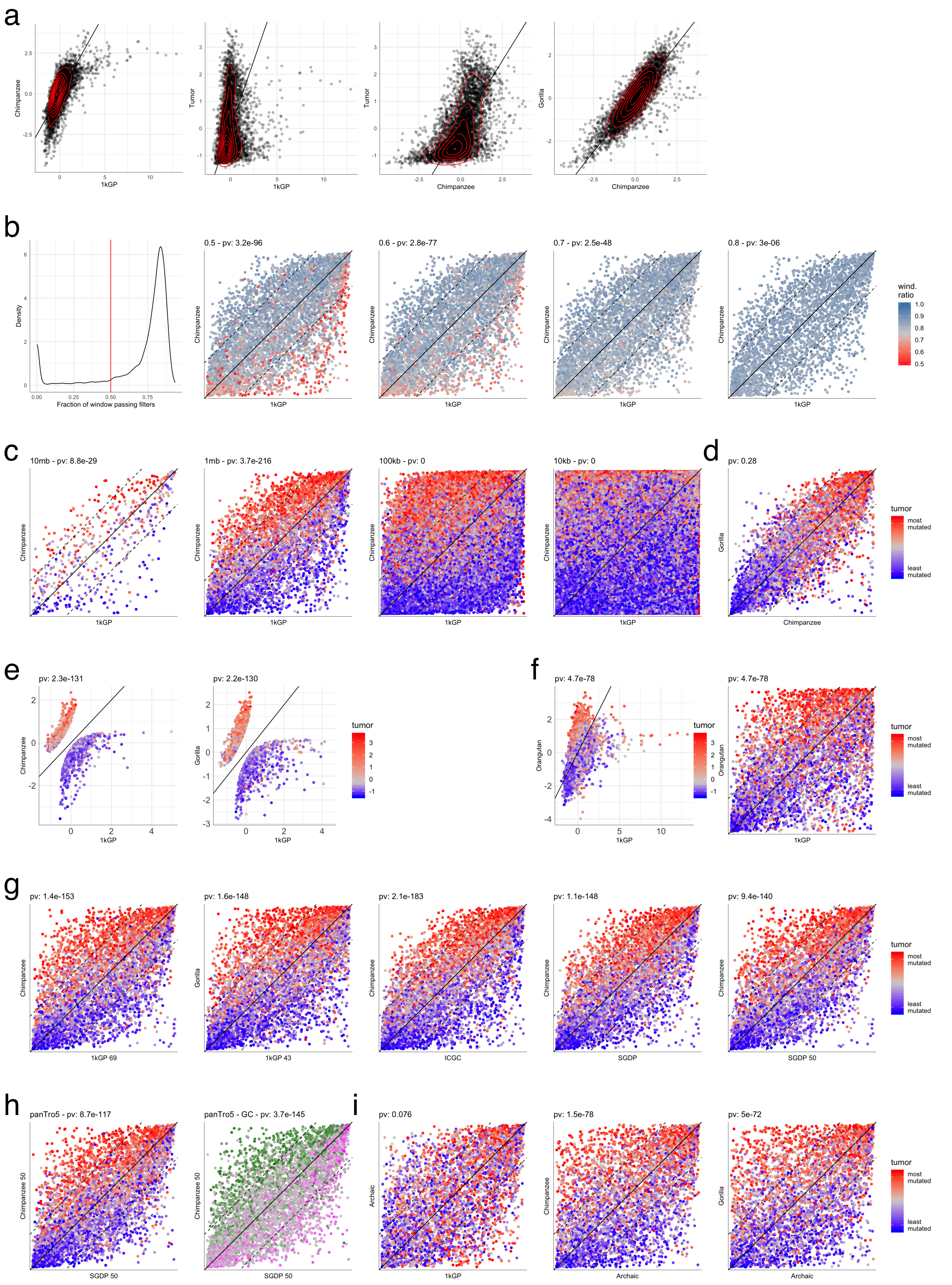

Supplementary Figure 1:

Robustness of the results. **a)** distribution and 2D-density of the unranked mutation density in pairwise comparisons between datasets; **b)** density and distribution of the fraction of the window passing our filters (color), setting thresholds at 50%, 60%, 70%, and 80%. The numbers above the figure denote the two-sided Mann-Whitney U-test p-value dividing the data by the diagonal; **c)** distribution of the ranked mutation density using 10Mbp, 1Mbp, 100kbp, and 10kbp window sizes. Color denotes tumor ranked mutation density; **d)** comparison of the ranked mutation density between chimpanzee and gorilla; **e)** distribution of the unranked mutation density when using only windows with ≥25% difference in ranking in both datasets; **f)** comparison of the standardized (left) and ranked (right) mutation density between human, orangutan, and tumor; **g)** comparison of the ranked mutation density between NHGA and different human datasets; **h)** between human and chimpanzee mapped to the chimpanzee panTro5 reference genome. Color denotes tumor ranked mutation density (left) and GC-content (right; green: low GC-content, magenta: high GC-content); **i)** comparison of 3 archaic samples with modern humans and NHGA. Color denotes tumor ranked mutation density.

Suppl. Figure 2 NHGA subspecies and genomic features

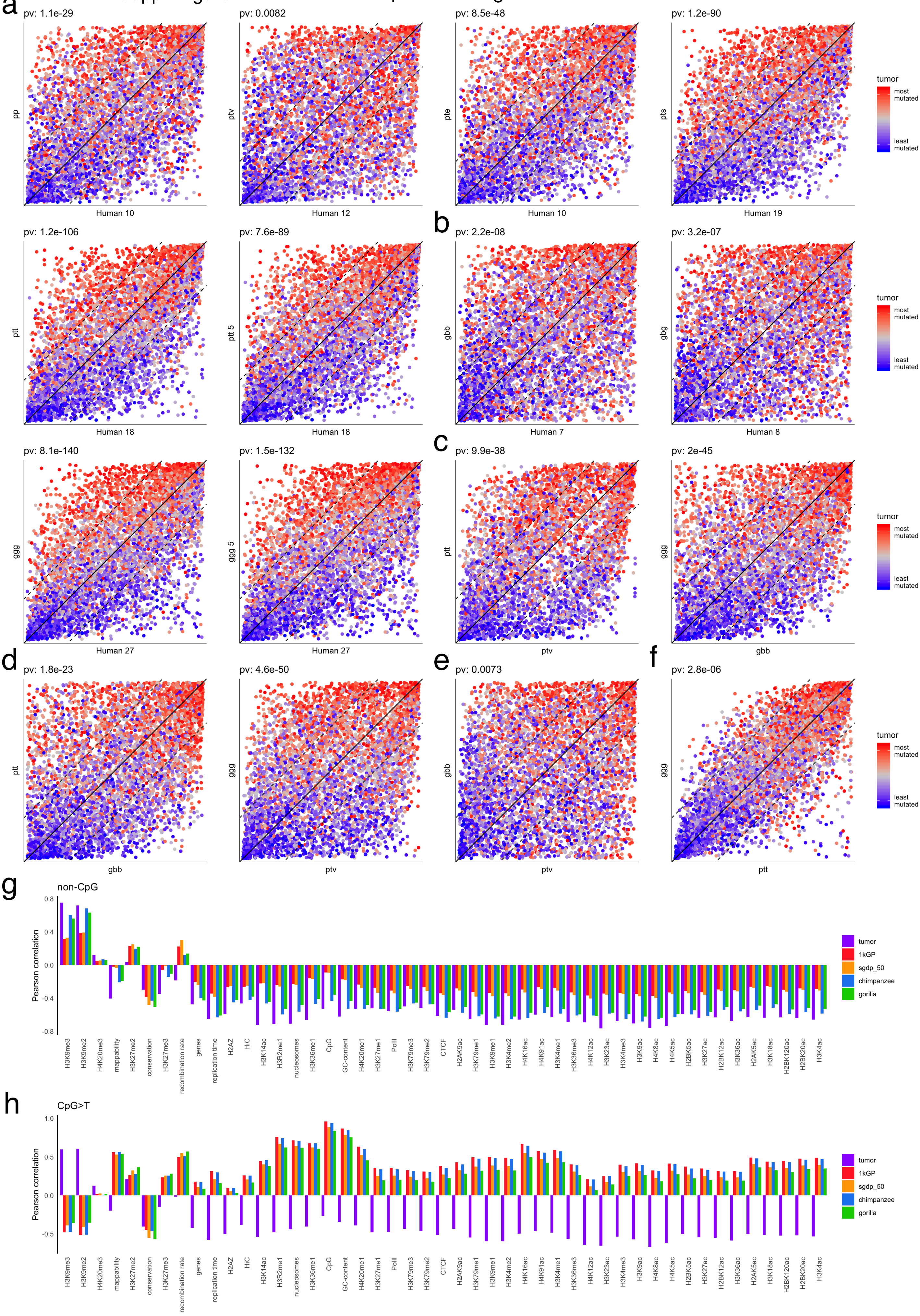

Supplementary Figure 2:

Subspecies. Comparisons of the ranked mutation density between human and NHGA subspecies. Color denotes tumor ranked mutation density. The list of subspecies names can be found in the legend of Supplementary Table 5. Human XX denotes a subset of XX 1kGP samples of African origin (to maximize human diversity) of the same sample size as the NHGA subspecies. The numbers above the figure denote the two-sided Mann-Whitney U-test p-value dividing the data by the diagonal. **a)** chimpanzee subspecies; **b)** gorilla subspecies; **c)** low-diversity (x-axis) vs high-diversity (y-axis) subspecies of the same species; **d)** low-diversity (x-axis) vs high-diversity (y-axis) subspecies of different NHGA species; **e)** low-diversity chimpanzee and gorillas; **f)** high-diversity chimpanzee and gorillas. **g)** Correlation (Pearson's R) between genomic and epigenomic features and mutations at non-CpG sites. Color denotes the different datasets; **h)** same as g, but using CpG>T transitions (not correcting by the sequence context).

Suppl. Figure 3 CpG–content and recombination

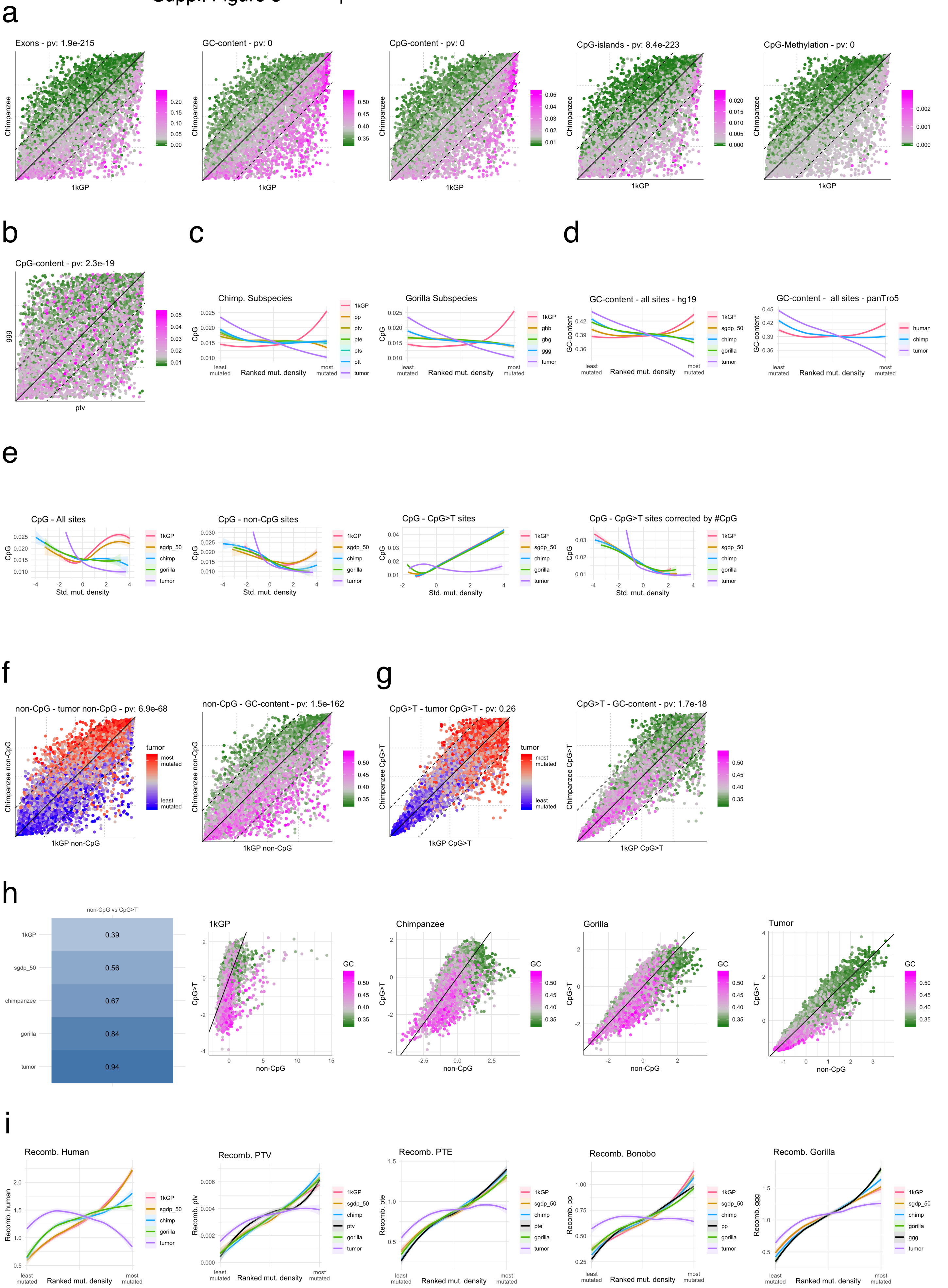

Supplementary Figure 3:

GC-content. **a)** Distribution of ranked mutation density in human and NHGA. Color denotes exons, GC-content, CpG-content, CpG-islands, and methylated CpG sites. The numbers above the figure denote the two-sided Mann-Whitney U-test p-value dividing the data by the diagonal; **b)** distribution of CpG-content (color) comparing a low-diversity chimpanzee (*Pan troglodytes* verus, x-axis) and a high-diversity gorilla (*Gorilla gorilla gorilla*, y-axis); **c)** loess smooth curves showing the association between ranked mutation density and CpG-content in NHGA subspecies. Color denotes dataset. Shaded area represents the 95% CI; **d)** association between GC-content in the mappings to the human and chimpanzee reference using all variants; **e)** association between CpG-content and the unranked mutation density (outliers were capped at ±4 standard deviations), using all sites, mutations at non-CpG sites, CpG>T transitions, and CpG>T transitions correcting by the number of CpG sites in the sequence; **f)** ranked distribution of non-CpG mutations in human and chimpanzee. Color denotes tumor ranked mutation density (left), and GC-content of the window (right). The numbers above the figure denote the two-sided Mann-Whitney U-test p-value dividing the data by the diagonal; **g)** same as f, but using the ranked distribution of CpG>T transitions (correcting by the fraction of CpG sites in the sequence) in human, chimpanzee, tumor, and GC-content; **h)** Pearson's correlation R (table color) and distribution (dots) between non-CpG and CpG>T (corrected by sequence content) mutation rate in each window within the same species. Dot color denotes GC-content; **i)** loess smooth curves showing the association between ranked mutation density and recombination rates in different species. Color denotes dataset. Shaded area represents the 95% CI. Black lines denote the exact subspecies from which the recombination data was obtained. Ptv: *Pan troglodytes verus*, Pte: *Pan troglodytes ellioti*, Pp: *Pan paniscus*, Ggg: *Gorilla gorilla gorilla*.

Trinucleotides

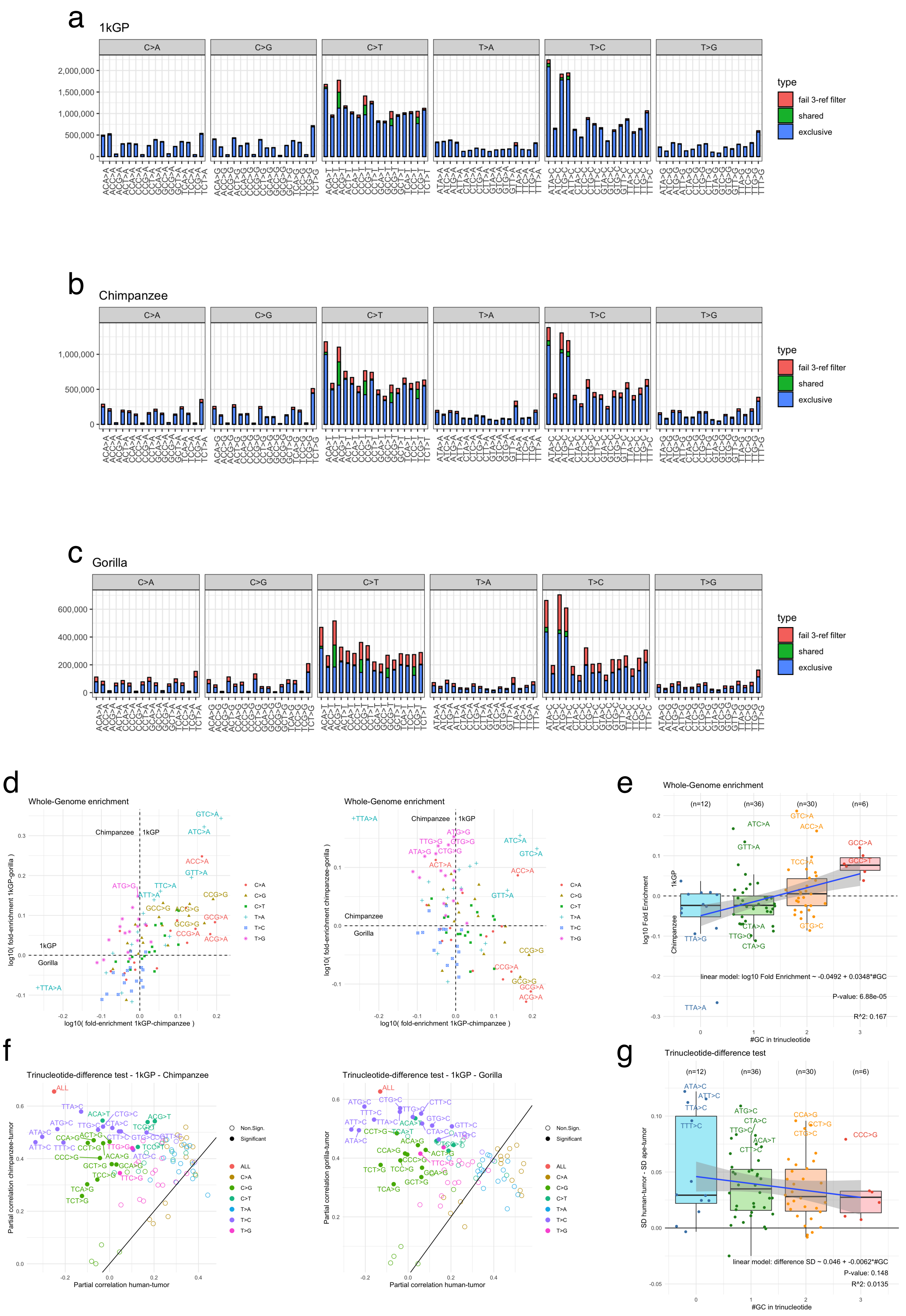

Supplementary Figure 4:

Trinucleotides. **a)** mutation spectrum (count of the raw number of mutations) of the 1kGP dataset. The colors denote the trinucleotides failing our 3-reference filter (see Methods), shared between 2 or more species, or species-exclusive; **b)** in chimpanzee; **c)** in gorilla; **d)** log10 fold-enrichment in 1kGP-chimpanzee vs 1kGP-gorilla (left), and 1kGP-chimpanzee vs chimpanzee-gorilla (right). Labels denote the top 10% most enriched and significant (enrichment test; chi-squared multiple testing p-value significance threshold $<5 \times 10^{-4}$, see Methods) trinucleotides in each axis. Color and dot shape denote the central nucleotide mutation; **e)** linear regression model showing a significant (two-sided F-test) association between the number of C and G bases within a trinucleotide (x axis) and the fold-enrichment in 1kGP-chimpanzee comparisons (y axis). The blue line denotes the linear model, and the shaded area the 95% CI. Box plots (center line, median; box limits, upper and lower quartiles; whiskers, 1.5× interquartile range, color: number of C and G bases in the trinucleotide) show all data points (including outliers) overlaid. The text inset contains the formula, coefficients, and p-value of the fitted linear model; **f)** trinucleotides-difference test (see Methods) comparing 1kGP-chimpanzee (left) and 1kGP-gorilla (right). The axes denote the partial correlation 1kGP-tumor (controlling by NHGA) and NHGA-tumor (controlling by 1kGP). Labels and filled circles denote significant trinucleotides with p-value $<10^{-5}$ (Kolmogorov-Smirnov test; multiple testing significance threshold $<5 \times 10^{-4}$). Color denotes the central nucleotide mutation; **g)** linear regression model test showing no significant association (two-sided F-test) between the trinucleotide-difference test effect size and the number of C and G bases in a trinucleotide, in the 1kGP-chimpanzee comparison. Colors, boxplots, and inset defined as in e.

Supplementary References:

1. Prado-Martinez, J. *et al.* Great ape genetic diversity and population history. *Nature* **499**, 471–5 (2013).
2. Xue, Y. *et al.* Mountain gorilla genomes reveal the impact of long-term population decline and inbreeding. *Science (80-.).* **348**, 242–245 (2015).
3. The 1000 Genomes Project Consortium. A global reference for human genetic variation. *Nature* **526**, 68–74 (2015).
4. Mallick, S. *et al.* The Simons Genome Diversity Project: 300 genomes from 142 diverse populations. *Nature* **538**, 201–206 (2016).
5. The ICGC/TCGA Pan-Cancer Analysis of Whole Genomes Consortium. Pan-cancer analysis of whole genomes. *Nature* **578**, 82–93 (2020).
6. Li, H. Aligning sequence reads, clone sequences and assembly contigs with BWA-MEM. Preprint at https://arxiv.org/abs/1303.3997 (2013).
7. McKenna, A. *et al.* The Genome Analysis Toolkit: A MapReduce framework for analyzing next-generation DNA sequencing data. *Genome Res.* **20**, 1297–1303 (2010).
8. DePristo, M. A. *et al.* A framework for variation discovery and genotyping using next-generation DNA sequencing data. *Nat. Genet.* **43**, 491–498 (2011).
9. Van der Auwera, G. A. *et al.* From FastQ data to high confidence variant calls: the Genome Analysis Toolkit best practices pipeline. *Curr. Protoc. Bioinforma.* **43**, 11.10.1-33 (2013).
10. Danecek, P. *et al.* The variant call format and VCFtools. *Bioinformatics* **27**, 2156–8 (2011).
11. Sudmant, P. H. *et al.* Evolution and diversity of copy number variation in the great ape lineage. *Genome Res.* **23**, 1373–82 (2013).
12. Hinrichs, A. S. *et al.* The UCSC Genome Browser Database: update 2006. *Nucleic Acids Res.* **34**, D590-8 (2006).
13. de Manuel, M. *et al.* Chimpanzee genomic diversity reveals ancient admixture with bonobos. *Science (80-.).* **354**, 477–481 (2016).
14. Benjamini, Y. & Speed, T. P. Summarizing and correcting the GC content bias in high-

throughput sequencing. *Nucleic Acids Res.* **40**, e72–e72 (2012).

15. Hodgkinson, A., Chen, Y. & Eyre-Walker, A. The large-scale distribution of somatic mutations in cancer genomes. *Hum. Mutat.* **33**, 136–143 (2012).

16. Tyekucheva, S. *et al.* Human-macaque comparisons illuminate variation in neutral substitution rates. *Genome Biol.* **9**, R76 (2008).

17. Ananda, G., Chiaromonte, F. & Makova, K. D. A genome-wide view of mutation rate co-variation using multivariate analyses. *Genome Biol.* **12**, R27 (2011).

18. Carlson, J. *et al.* Extremely rare variants reveal patterns of germline mutation rate heterogeneity in humans. *Nat. Commun.* **9**, 3753 (2018).

19. Polak, P. *et al.* Cell-of-origin chromatin organization shapes the mutational landscape of cancer. *Nature* **518**, 360–364 (2015).

20. Alexandrov, L. B. *et al.* The repertoire of mutational signatures in human cancer. *Nature* **578**, 94–101 (2020).

21. García-Nieto, P. E. *et al.* Carcinogen susceptibility is regulated by genome architecture and predicts cancer mutagenesis. *EMBO J.* **36**, 2829–2843 (2017).

22. Giannoulatou, E. *et al.* Whole-genome sequencing of spermatocytic tumors provides insights into the mutational processes operating in the male germline. *PLoS One* **12**, e0178169 (2017).

23. Dorssers, L. C. J. *et al.* Molecular heterogeneity and early metastatic clone selection in testicular germ cell cancer development. *Br. J. Cancer* **120**, 444–452 (2019).

24. Li, C. H., Haider, S., Shiah, Y.-J., Thai, K. & Boutros, P. C. Sex Differences in Cancer Driver Genes and Biomarkers. *Cancer Res.* **78**, 5527–5537 (2018).

25. Tomasetti, C., Li, L. & Vogelstein, B. Stem cell divisions, somatic mutations, cancer etiology, and cancer prevention. *Science (80-.).* **355**, 1330–1334 (2017).

26. Podolskiy, D. I. *et al.* Analysis of cancer genomes reveals basic features of human aging and its role in cancer development. *Nat. Commun.* **7**, 12157 (2016).

27. Klebanov, N. *et al.* Burden of unique and low prevalence somatic mutations correlates with cancer survival. *Sci. Rep.* **9**, 4848 (2019).

28. García-Nieto, P. E., Morrison, A. J. & Fraser, H. B. The somatic mutation landscape of the human body. *Genome Biol.* **20**, 298 (2019).

29. Osorio, F. G. *et al.* Somatic Mutations Reveal Lineage Relationships and Age-Related Mutagenesis in Human Hematopoiesis. *Cell Rep.* **25**, 2308-2316.e4 (2018).

30. Prüfer, K. *et al.* The complete genome sequence of a Neanderthal from the Altai Mountains. *Nature* **505**, 43–9 (2014).

31. Prüfer, K. *et al.* A high-coverage Neandertal genome from Vindija Cave in Croatia. *Science* **358**, 655–658 (2017).

32. Meyer, M. *et al.* A high-coverage genome sequence from an archaic Denisovan individual. *Science* **338**, 222–6 (2012).

33. Nater, A. *et al.* Morphometric, Behavioral, and Genomic Evidence for a New Orangutan Species. *Curr. Biol.* **27**, 3487-3498.e10 (2017).

34. Lercher, M. J. & Hurst, L. D. Human SNP variability and mutation rate are higher in regions of high recombination. *Trends Genet.* **18**, 337–340 (2002).

35. Hellmann, I., Ebersberger, I., Ptak, S. E., Pääbo, S. & Przeworski, M. A Neutral Explanation for the Correlation of Diversity with Recombination Rates in Humans. *Am. J. Hum. Genet.* **72**, 1527–1535 (2003).

36. Hellmann, I. *et al.* Why do human diversity levels vary at a megabase scale? *Genome Res.* **15**, 1222–31 (2005).

37. Schuster-Böckler, B. & Lehner, B. Chromatin organization is a major influence on regional mutation rates in human cancer cells. *Nature* **488**, 504–507 (2012).

38. Kong, A. *et al.* A high-resolution recombination map of the human genome. *Nat. Genet.* **31**, 241–247 (2002).

39. Pratto, F. *et al.* DNA recombination. Recombination initiation maps of individual human genomes. *Science* **346**, 1256442 (2014).

40. Auton, A. *et al.* A fine-scale chimpanzee genetic map from population sequencing. *Science (80-.).* **336**, 193–8 (2012).

41. Stevison, L. S. *et al.* The Time Scale of Recombination Rate Evolution in Great Apes. *Mol. Biol. Evol.* **33**, 928–945 (2016).

42. Kübler, K. *et al.* Tumor mutational landscape is a record of the pre-malignant state. Preprint at https://www.biorxiv.org/content/10.1101/517565v1 (2019).

43. Pollard, K. S., Hubisz, M. J., Rosenbloom, K. R. & Siepel, A. Detection of nonneutral

substitution rates on mammalian phylogenies. *Genome Res.* **20**, 110–21 (2010).

44. Ernst, J. *et al.* Mapping and analysis of chromatin state dynamics in nine human cell types. *Nature* **473**, 43–49 (2011).

45. Zoubak, S., Clay, O. & Bernardi, G. The gene distribution of the human genome. *Gene* **174**, 95–102 (1996).

46. International Human Genome Sequencing Consortium. Initial sequencing and analysis of the human genome. *Nature* **409**, 860–921 (2001).

47. Amit, M. *et al.* Differential GC content between exons and introns establishes distinct strategies of splice-site recognition. *Cell Rep.* **1**, (2012).

48. Hwang, D. G. & Green, P. Bayesian Markov chain Monte Carlo sequence analysis reveals varying neutral substitution patterns in mammalian evolution. *Proc. Natl. Acad. Sci. U. S. A.* **101**, 13994–4001 (2004).

49. Keightley, P. D., Eöry, L., Halligan, D. L. & Kirkpatrick, M. Inference of Mutation Parameters and Selective Constraint in Mammalian Coding Sequences by Approximate Bayesian Computation. *Genetics* **187**, 1153–1161 (2011).

50. Siepel, A. & Haussler, D. Phylogenetic Estimation of Context-Dependent Substitution Rates by Maximum Likelihood. *Mol. Biol. Evol.* **21**, 468–488 (2003).

51. Nachman, M. W. & Crowell, S. L. Estimate of the Mutation Rate per Nucleotide in Humans. *Genetics* **156**, 297–304 (2000).

52. ENCODE Project Consortium. An integrated encyclopedia of DNA elements in the human genome. *Nature* **489**, 57–74 (2012).

53. Hernando-Herraez, I. *et al.* Dynamics of DNA Methylation in Recent Human and Great Ape Evolution. *PLoS Genet.* **9**, e1003763 (2013).

54. Mugal, C. F. & Ellegren, H. Substitution rate variation at human CpG sites correlates with non-CpG divergence, methylation level and GC content. *Genome Biol.* **12**, R58 (2011).

55. Acuna-Hidalgo, R., Veltman, J. A. & Hoischen, A. New insights into the generation and role of de novo mutations in health and disease. *Genome Biol.* **17**, 241 (2016).

56. Vavouri, T. & Lehner, B. Human genes with CpG island promoters have a distinct transcription-associated chromatin organization. *Genome Biol.* **13**, R110 (2012).

57. Santoni, F. A. EMdeCODE: a novel algorithm capable of reading words of epigenetic code to predict enhancers and retroviral integration sites and to identify H3R2me1 as a distinctive mark of coding versus non-coding genes. *Nucleic Acids Res.* **41**, e48 (2013).
58. Rimmer, A. *et al.* Integrating mapping-, assembly- and haplotype-based approaches for calling variants in clinical sequencing applications. *Nat. Genet.* **46**, 912–918 (2014).
59. Lek, M. *et al.* Analysis of protein-coding genetic variation in 60,706 humans. *Nature* **536**, 285–291 (2016).
60. Besenbacher, S., Hvilsom, C., Marques-Bonet, T., Mailund, T. & Schierup, M. H. Direct estimation of mutations in great apes reconciles phylogenetic dating. *Nat. Ecol. Evol.* **3**, 286–292 (2019).
61. Jónsson, H. *et al.* Parental influence on human germline de novo mutations in 1,548 trios from Iceland. *Nature* **549**, 519–522 (2017).
62. Aggarwala, V. & Voight, B. F. An expanded sequence context model broadly explains variability in polymorphism levels across the human genome. **48**, (2016).
63. Wong, W. S. W. *et al.* New observations on maternal age effect on germline de novo mutations. *Nat. Commun.* **7**, 10486 (2016).
64. Rahbari, R. *et al.* Timing, rates and spectra of human germline mutation. *Nat. Genet.* **48**, 126–133 (2016).
65. Francioli, L. C. *et al.* Genome-wide patterns and properties of de novo mutations in humans. *Nat. Genet.* **47**, 822–826 (2015).
66. Moorjani, P., Amorim, C. E. G., Arndt, P. F. & Przeworski, M. Variation in the molecular clock of primates. *Proc. Natl. Acad. Sci.* **113**, 10607–10612 (2016).
67. Smith, T. C. A., Arndt, P. F. & Eyre-Walker, A. Large scale variation in the rate of germ-line de novo mutation, base composition, divergence and diversity in humans. *PLOS Genet.* **14**, e1007254 (2018).
68. Tate, J. G. *et al.* COSMIC: the Catalogue Of Somatic Mutations In Cancer. *Nucleic Acids Res.* **47**, D941–D947 (2019).
69. Alexandrov, L. B. *et al.* Clock-like mutational processes in human somatic cells. *Nat. Genet.* **47**, 1402–1407 (2015).
70. Svardal, H. *et al.* Ancient hybridization and strong adaptation to viruses across African

vervet monkey populations. *Nat. Genet.* **49**, 1705–1713 (2017).

71. Harr, B. *et al.* Genomic resources for wild populations of the house mouse, Mus musculus and its close relative Mus spretus. *Sci. data* **3**, 160075 (2016).

72. Groenen, M. A. M. *et al.* Analyses of pig genomes provide insight into porcine demography and evolution. *Nature* **491**, 393–398 (2012).

73. Harris, K. & Pritchard, J. K. Rapid evolution of the human mutation spectrum. *Elife* **6**, (2017).

74. Blokzijl, F. *et al.* Tissue-specific mutation accumulation in human adult stem cells during life. *Nature* **538**, 260–264 (2016).

75. Martincorena, I. *et al.* High burden and pervasive positive selection of somatic mutations in normal human skin. *Science (80-.).* **348**, 880–6 (2015).
