# Supplementary material for: Extreme differences between human germline and tumor mutation densities are driven by ancestral human-specific deviations
Source: Nat Commun. 2020 May 19;11:2512. doi: 10.1038/s41467-020-16296-4 (PMC7237693; doi:10.1038/s41467-020-16296-4)
Supplement: Supplementary file 3 — Description of Additional Supplementary Files [file 41467_2020_16296_MOESM3_ESM.docx]

**Description of Additional Supplementary Files**

**File name:** Supplementary Data 1

**Description:** Trinucleotide-difference test in tumor types. Difference between the standard deviations of the distributions in the trinucleotide-difference test using individual tumor types comparing 1kGP-chimpanzee (page 1), and 1kGP-gorilla (page 2). Only results with p-value <10-5 are shown (Kolmogorov-Smirnov test; multiple testing significance threshold <5x10-4).
