# Supplementary material for: Extreme differences between human germline and tumor mutation densities are driven by ancestral human-specific deviations
Source: Nat Commun. 2020 May 19;11:2512. doi: 10.1038/s41467-020-16296-4 (PMC7237693; doi:10.1038/s41467-020-16296-4)
Supplement: Supplementary file 5 — Reporting Summary [file 41467_2020_16296_MOESM5_ESM.pdf]

nature research

Corresponding author(s): Arcadi Navarro & David Juan

Last updated by author(s): Apr 6, 2020

Reporting Summary

Nature Research wishes to improve the reproducibility of the work that we publish. This form provides structure for consistency and transparency in reporting. For further information on Nature Research policies, see Authors & Referees and the Editorial Policy Checklist .

Statistics

For all statistical analyses, confirm that the following items are present in the figure legend, table legend, main text, or Methods section.

n/a	Confirmed	
☐	☒	The exact sample size (*n*) for each experimental group/condition, given as a discrete number and unit of measurement
☒	☐	A statement on whether measurements were taken from distinct samples or whether the same sample was measured repeatedly
☐	☒	The statistical test(s) used AND whether they are one- or two-sided<br>*Only common tests should be described solely by name; describe more complex techniques in the Methods section.*
☒	☐	A description of all covariates tested
☐	☒	A description of any assumptions or corrections, such as tests of normality and adjustment for multiple comparisons
☐	☒	A full description of the statistical parameters including central tendency (e.g. means) or other basic estimates (e.g. regression coefficient) AND variation (e.g. standard deviation) or associated estimates of uncertainty (e.g. confidence intervals)
☐	☒	For null hypothesis testing, the test statistic (e.g. *F*, *t*, *r*) with confidence intervals, effect sizes, degrees of freedom and *P* value noted<br>*Give P values as exact values whenever suitable.*
☒	☐	For Bayesian analysis, information on the choice of priors and Markov chain Monte Carlo settings
☒	☐	For hierarchical and complex designs, identification of the appropriate level for tests and full reporting of outcomes
☐	☒	Estimates of effect sizes (e.g. Cohen's *d*, Pearson's *r*), indicating how they were calculated

Our web collection on statistics for biologists contains articles on many of the points above.

Software and code

Policy information about availability of computer code

Data collection	No data collection software was used in this study
Data analysis	Sequence mapping: BWA MEM v0.7.15<br>Variant Calling: GATK v3.7<br>Filtering: VCFTOOLS v0.1.12, BEDTOOLS v2.26.0, UCSCtools v331 (LiftOver tool), Seqbility v20091110 (SNPable regions)<br>Processing: user-made scripts in BASH v4.1.2<br>Statistical Analysis: R v3.6.0

For manuscripts utilizing custom algorithms or software that are central to the research but not yet described in published literature, software must be made available to editors/reviewers. We strongly encourage code deposition in a community repository (e.g. GitHub). See the Nature Research guidelines for submitting code & software for further information.

Data

Policy information about availability of data

All manuscripts must include a data availability statement. This statement should provide the following information, where applicable:

- Accession codes, unique identifiers, or web links for publicly available datasets
- A list of figures that have associated raw data
- A description of any restrictions on data availability

All the analyses in this study were performed using publicly available data.

Human datasets:
1000 Genomes Project: ftp://ftp.1000genomes.ebi.ac.uk/vol1/ftp/release/20130502/
Simons Genome Diversity Project: EBI European Nucleotide Archive (accession numbers PRJEB9586 and ERP010710)

International Cancer Genome Consortium (https://dcc.icgc.org/releases/PCAWG/germline_variations)
de novo mutations: European Variant Archive (accession number PRJEB15197)

Non-human Great Ape datasets:
The Great Apes Genome Project: Sequence Read Archive (SRA) (PRJNA189439 and SRP018689). European Nucleotide Archive (ENA) accession numbers PRJEB15086 (Chimpanzee), PRJEB3220 (Gorilla), and PRJEB19688 (Orangutan)

Tumor datasets:
Pan-Cancer Analysis of Whole Genomes: https://dcc.icgc.org/pcawg/

Archaic samples (http://cdna.eva.mpg.de/neandertal/Vindija/)

Other datasets used are properly referenced in the manuscript and in the supplementary notes

Field-specific reporting

Please select the one below that is the best fit for your research. If you are not sure, read the appropriate sections before making your selection.

- [x] Life sciences
- [ ] Behavioural & social sciences
- [ ] Ecological, evolutionary & environmental sciences

For a reference copy of the document with all sections, see nature.com/documents/nr-reporting-summary-flat.pdf

Life sciences study design

All studies must disclose on these points even when the disclosure is negative.

Sample size
We used all saples available in each dataset. We tested the robustness of the results using subsets of the human datasets matching the smaller sample size of the non-human primates datasets and obtained virtually identical results.

Data exclusions
One gorilla sample (Serufuli) was excluded from the analysis because its FASTQ files were incomplete/malformed in the repository at the moment of accession.

Replication
We repeated the main analysis using different human datasets. The results were replicated in all occasions. Through the manuscript we use two human datasets (1kGP and sgdp_50) with very different sample sizes, and their results are always consistent.

Analysis of trinucleotides showed incoherences in CpG>T sites between the two human datasets with different sample sizes, as indicated in the main text and supplementary notes. These incoherences were expected and consistent with the nature of the CpG>T mutations. The smaller human dataset was not used for the trinucleotide-difference test nor the signature-difference test, due to its lack of power when dividing its smaller number of SNVs across 5,040 1Mbp windows.

Randomization
Sample randomization was not applicable given the nature of our comparative analyses focused on large datasets. Instead, we performed parametric significance tests corrected by multiple testing when applicable. Additionally, we repeated the analyses for different datasets and informative subsamples under different conditions, and we extensively investigated the possible technical and biological contributors to our observations.

Blinding
Blinding was not relevant to our study. Our study is a comparative analysis based on data from previously published large scale datasets and the data retrieved from them was not subjective.

Reporting for specific materials, systems and methods

We require information from authors about some types of materials, experimental systems and methods used in many studies. Here, indicate whether each material, system or method listed is relevant to your study. If you are not sure if a list item applies to your research, read the appropriate section before selecting a response.

Materials & experimental systems

n/a	Involved in the study
[x]	[ ] Antibodies
[x]	[ ] Eukaryotic cell lines
[x]	[ ] Palaeontology
[x]	[ ] Animals and other organisms
[x]	[ ] Human research participants
[x]	[ ] Clinical data

Methods

n/a	Involved in the study
[x]	[ ] ChIP-seq
[x]	[ ] Flow cytometry
[x]	[ ] MRI-based neuroimaging
